# Global, regional and national burden of colorectal cancer attributable to low-fiber diet from 1990 to 2021: a systematic analysis of the global burden of disease study 2021

**DOI:** 10.3389/fnut.2026.1688108

**Published:** 2026-06-04

**Authors:** Chao Ma, Bing Yan, Ge Li, Yongsheng Jiang

**Affiliations:** 1 Department of Gastrointestinal Surgery, Central Hospital Affiliated to Shandong First Medical University, Jinan Central Hospital, Jinan, Shandong, China; 2 School of Clinical Medicine, Hebei University, Affiliated Hospital of Hebei University, Baoding, Hebei, China

**Keywords:** colorectal cancer, DALYs, GBD 2021, health inequality, low-fiber diet, SDI

## Abstract

**Background:**

Low-fiber diet is a modifiable dietary risk factor for colorectal cancer (CRC), but its long-term attributable burden and socioeconomic disparities remain insufficiently characterized. This study aimed to assess the global, regional, and national burden of colorectal cancer attributable to low-fiber diet (CRC-LFD) from 1990 to 2021 and to project future trends.

**Methods:**

Data were obtained from the Global Burden of Disease Study 2021. The estimation of CRC-LFD burden was based on the GBD comparative risk assessment framework, which integrates population-level exposure distributions, theoretical minimum risk exposure levels, and relative risks derived from meta-analyses to calculate population attributable fractions. Deaths, disability-adjusted life years (DALYs), age-standardized mortality rates (ASMRs), and age-standardized DALY rates (ASDRs) were analyzed. The Socio-demographic Index (SDI) was used to assess burden patterns across development levels. Das Gupta decomposition was applied to quantify the contributions of population growth, population aging, and epidemiological change. Cross-national inequality was evaluated using the Slope Index of Inequality and Concentration Index. Future trends to 2045 were projected using the Nordpred model.

**Results:**

From 1990 to 2021, global DALYs and deaths attributable to CRC-LFD increased by 24 and 36%, respectively, whereas ASDR and ASMR declined. The largest absolute DALY burden occurred in middle SDI regions, while the highest ASDR and ASMR were observed in high SDI regions. Among individuals younger than 80 years, males had higher attributable DALYs and deaths than females. Inequality analyses indicated persistent socioeconomic disparities, with relative inequality showing an increasing trend. Decomposition analysis suggested that, despite declining age-standardized rates, the absolute burden continued to rise mainly because of demographic changes, particularly population growth and aging. Projections indicated that deaths and DALYs would continue to increase by 2045, although ASDR and ASMR were expected to decline.

**Conclusion:**

CRC-LFD shows a paradoxical pattern of declining age-standardized rates but increasing absolute burden. These findings highlight the need to incorporate dietary fiber promotion into comprehensive CRC prevention strategies, develop systematic dietary policies, and implement SDI-specific interventions. As an ecological study, the results should be interpreted at the population level and cannot establish individual-level causality.

## Introduction

Colorectal cancer (CRC) is one of the most common malignancies worldwide and remains a major contributor to cancer-related morbidity and mortality (1–3). Its burden is shaped by demographic expansion, population aging, socioeconomic transition, and exposure to modifiable lifestyle and dietary risk factors. CRC arises from the colon and rectum and corresponds to ICD-10 codes C18–C20 (4). Although advances in screening, diagnosis, and treatment have improved outcomes in some settings, substantial disparities persist across countries, sexes, age groups, and levels of socioeconomic development (5). Men generally experience higher CRC incidence and mortality than women, reflecting the combined influence of biological susceptibility, behavioral exposures, and differences in preventive health service utilization (6, 7). Among modifiable determinants, diet has received increasing attention because it may influence CRC risk at both the individual and population levels.

Low dietary fiber intake is an established dietary risk factor for CRC (8). Evidence from prospective studies and dose–response meta-analyses has shown an inverse association between dietary fiber intake and CRC risk (9). For example, a meta-analysis reported that each 10 g/day increase in total dietary fiber intake was associated with a 10% lower risk of CRC (10). Similar protective associations have been reported for cereal fiber and whole-grain intake, supporting the role of fiber-rich foods in CRC prevention (11). However, inadequate fiber consumption remains common worldwide, and average intake in many populations remains below commonly recommended levels (12). This creates a persistent gap between dietary recommendations and actual population exposure, making low-fiber diet (LFD) an important target for prevention-oriented burden assessment.

The biological plausibility linking LFD to CRC is supported by several interrelated mechanisms. Dietary fiber increases stool bulk, shortens colonic transit time, and may reduce mucosal exposure to carcinogens (13). More importantly, fiber serves as a major substrate for gut microbial fermentation, leading to the production of short-chain fatty acids, particularly butyrate (14). Butyrate contributes to epithelial barrier integrity, regulation of cell proliferation and apoptosis, anti-inflammatory signaling, and maintenance of intestinal immune homeostasis (15). Insufficient fiber intake may therefore promote gut microbiota dysbiosis, reduce butyrate generation, impair mucosal defense, and favor chronic low-grade inflammation, thereby creating a biological environment conducive to colorectal carcinogenesis (16). These mechanisms provide a biologically coherent explanation for the population-level observation that CRC risk is higher in settings where fiber intake is insufficient, particularly when combined with other dietary and metabolic risk factors (17).

Despite strong epidemiological and mechanistic evidence, several scientific questions remain insufficiently clarified. First, most previous studies have evaluated the association between dietary fiber intake and CRC risk at the individual level, whereas fewer studies have quantified the population-level disease burden specifically attributable to LFD across countries and regions (18). Second, broader analyses of dietary risk factors have often examined multiple dietary exposures together, such as low intake of whole grains, milk, calcium, or high intake of red and processed meat, but they may not fully characterize the specific geographic, socioeconomic, age-, and sex-specific patterns of CRC burden attributable to LFD alone (19). Therefore, the long-term global distribution, inequality pattern, demographic drivers, and future trajectory of colorectal cancer attributable to low-fiber diet (CRC-LFD) remain incompletely understood.

The Global Burden of Disease Study provides a suitable framework to address this gap because it offers standardized and comparable estimates of disease burden by cause, risk factor, age, sex, location, and year (20–22). The GBD framework enables the estimation of deaths, disability-adjusted life years (DALYs), age-standardized rates, population-attributable fractions, and uncertainty intervals across countries and development strata, thereby allowing cross-national and temporal comparisons using a consistent methodological structure (23). Nevertheless, GBD estimates should be interpreted cautiously because they depend on available input data, modeled exposure distributions, relative-risk assumptions, and counterfactual exposure definitions; therefore, they are best used to describe comparative burden and generate public health evidence rather than to infer individual-level causality.

Using data from GBD 2021, this study aimed to quantify the global, regional, and national burden of CRC-LFD from 1990 to 2021. Specifically, we assessed temporal trends in deaths and DALYs, characterized age-, sex-, and Socio-demographic Index (SDI)-specific patterns, examined cross-national inequalities, decomposed changes in burden into demographic and epidemiological components, and projected future trends through 2045. By focusing specifically on CRC-LFD and integrating trend analysis, inequality assessment, decomposition, and projection, this study seeks to clarify how insufficient fiber intake contributes to CRC burden across heterogeneous populations and to provide evidence for dietary prevention strategies and targeted resource allocation.

## Methods

### Data source and study design

Data were obtained from the Global Burden of Disease Study 2021 (GBD 2021), which provides comparable estimates of disease burden for 204 countries and territories from 1990 to 2021 (24). Estimates were derived from multiple data sources, including vital registration systems, cancer registries, dietary surveys, and epidemiological studies, using standardized GBD modeling frameworks (25). All estimates were reported with 95% uncertainty intervals (UIs), generated from 1,000 posterior draws.

Data on CRC-LFD were downloaded from the Global Health Data Exchange (GHDx) query tool. We selected “Diet low in fiber” as the risk factor, “Colon and rectum cancer” as the cause, deaths and DALYs as measures, number and rate as metrics, and included global, SDI regions, and 204 countries and territories from 1990 to 2021. This study followed the GATHER guidelines (26).

### Data processing

Data were obtained from the GHDx as aggregated GBD 2021 estimates. We conducted secondary processing only after data download, including merging datasets by location, year, sex, age group, measure, metric, cause, and risk factor; extracting point estimates and 95% uncertainty interval (UI) bounds; calculating percentage changes; and reshaping data for trend, decomposition, inequality, and projection analyses (23). No individual-level data were used. We did not re-estimate the original GBD models, exposure distributions, relative risks, or population-attributable fractions; all burden estimates were taken directly from GBD 2021.

### Definition of low-fiber diet

In GBD, LFD is defined as mean daily dietary fiber intake below the theoretical minimum risk exposure level (TMREL), approximately 23–25 g/day (27). CRC-LFD refers to the CRC burden statistically attributable to suboptimal fiber intake. Dietary fiber exposure was estimated from population-level dietary data and modeled using spatiotemporal Gaussian process regression. However, dietary assessment may be affected by recall bias, survey heterogeneity, food composition differences, and cultural variation in dietary patterns. Therefore, LFD in this study should be interpreted as a standardized GBD exposure definition rather than direct individual-level dietary measurement.

### Estimation of attributable burden

GBD estimates risk-attributable burden using the comparative risk assessment framework. The population attributable fraction (PAF) represents the proportion of disease burden that would theoretically be avoided if the observed exposure distribution were shifted to the TMREL (27). For dietary fiber, PAF estimation depends on three key elements: observed fiber intake distribution, relative risk estimates linking fiber intake to CRC, and the TMREL. Attributable deaths and DALYs were calculated by applying PAFs to the total CRC burden. In this study, we directly used GBD 2021 CRC-LFD estimates rather than recalculating PAFs.

### Socio-demographic index

The Socio-demographic Index (SDI) is a composite indicator developed and provided by the Global Burden of Disease Study to summarize the overall level of social and economic development for each location in each year (24). It is calculated based on three components: lag-distributed income per capita, average educational attainment among individuals aged 15 years and older, and the total fertility rate among individuals younger than 25 years. SDI ranges from 0 to 1, with higher values indicating higher sociodemographic development. In this study, SDI values and SDI-based regional classifications were obtained directly from the GBD 2021 database through the Global Health Data Exchange (GHDx) results tool. According to the GBD classification, countries and territories were grouped into five SDI categories: low, low-middle, middle, high-middle, and high SDI. SDI was used to describe socioeconomic patterns and cross-national inequalities in CRC-LFD burden.

### Trend analysis

Temporal trends in age-standardized mortality rate (ASMR) and age-standardized DALY rate (ASDR) were assessed using estimated annual percentage change (EAPC). A log-linear regression model was fitted: ln(rate) = *α* + *β* × calendar year + *ε* (28). EAPC was calculated as: EAPC = 100 × (e^β^ − 1). An EAPC greater than 0 indicates an increasing trend, whereas an EAPC less than 0 indicates a decreasing trend. This method assumes that the logarithm of the age-standardized rate changes approximately linearly over time. If trends are nonlinear or affected by abrupt changes in data quality, policy, screening, or health systems, EAPC may oversimplify the temporal pattern. Therefore, EAPC was interpreted as an average long-term trend indicator.

### Decomposition analysis

The Das Gupta decomposition method was used to separate changes in absolute deaths and DALYs into three components: population growth, population aging, and epidemiological change (29). Population growth reflects changes in total population size; population aging reflects changes in age structure; and epidemiological change reflects changes in age-specific burden rates after accounting for demographic shifts. This method was selected because it provides a clear additive framework for distinguishing demographic drivers from changes in disease risk. However, the epidemiological component may reflect multiple factors, including dietary exposure, diagnosis, treatment, survival, and GBD modeling assumptions, and should not be interpreted as a single causal mechanism.

### Cross-national inequality analysis

Cross-national inequality was assessed using the Slope Index of Inequality (SII) and Concentration Index (CI). SII reflects absolute inequality across the SDI distribution (21). A positive SII indicates that CRC-LFD burden is higher in higher-SDI countries, whereas a negative SII indicates a higher burden in lower-SDI countries. CI reflects relative inequality and ranges from −1 to +1. A positive CI suggests that the burden is concentrated in high-SDI countries, while a negative CI suggests concentration in low-SDI countries. Values close to 0 indicate a relatively even distribution. In this study, SII and CI were used as descriptive measures of cross-national inequality rather than as indicators of causal association. Because formal sensitivity analyses excluding extreme values, applying alternative population-weighting schemes, or using alternative socioeconomic rankings were not performed, changes in SII and CI should be interpreted cautiously as descriptive patterns of inequality. Statistical significance was not inferred solely from numerical changes in these indices.

### Forecasting analysis

Future deaths and DALYs attributable to CRC-LFD were projected using the Nordpred model, which is based on an age-period-cohort framework (30). The model uses historical age-specific rates and population projections to estimate future burden. Nordpred was selected because it is commonly used for cancer burden projection and can incorporate age, period, and cohort effects. However, its projections assume that past trends will partially continue, and may be affected by future changes in screening, treatment, diet, prevention strategies, and population structure.

### Limitations of GBD methodology

Several methodological limitations should be noted. First, GBD estimates are model-based and depend on the quality and availability of input data, which vary across countries and years. Second, dietary fiber intake is difficult to measure accurately and may be affected by recall bias, dietary survey differences, and regional dietary culture. Third, risk-attributable estimates rely on relative risk functions from epidemiological studies and may be influenced by residual confounding. Fourth, PAF-based estimates represent theoretical avoidable burden under a counterfactual exposure scenario and should not be interpreted as directly preventable burden in real-world settings. Finally, EAPC, Das Gupta decomposition, SII, CI, and Nordpred each rely on specific statistical assumptions, which should be considered when interpreting the results.

## Results

### Global and regional burden of CRC-LFD

Globally, the number of DALYs attributable to CRC-LFD increased from 247,015.19 (95% UI: 112,606.13 to 380,299.24) in 1990 to 305,675.95 (95% UI: 135,088.79 to 469,863.48) in 2021, representing a 24% increase. Similarly, deaths increased from 9,689.21 (95% UI: 4,409.65 to 14,808.08) to 13,144.85 (95% UI: 5,761.65 to 20,265.10), corresponding to a 36% increase. However, age-standardized rates declined over the same period. In 2021, the ASDR and ASMR were 3.58 (95% UI: 1.58 to 5.50) and 0.16 (95% UI: 0.07 to 0.24) per 100,000 population, respectively, both markedly lower than in 1990 [6.17 (95% UI: 2.80 to 9.47) and 0.27 (95% UI: 0.12 to 0.42)]. The EAPCs were −1.89 (95% CI: −1.95 to −1.83) for ASDR and −1.86 (95% CI: −1.90 to −1.82) for ASMR, indicating sustained downward trends in age-standardized burden (Tables 1, 2). These divergent trends suggest that the global increase in absolute DALYs and deaths was mainly driven by population growth and aging, whereas the age-specific burden of CRC-LFD may have improved over time.

**Table 1:** Disability-adjusted life years (DALYs) and age-standardized DALY rates (ASDR) of colorectal cancer attributable to low-fiber diet (CRC-LFD) in 1990 and 2021, with percentage change (PC) and estimated annual percentage change (EAPC) from 1990 to 2021.

Location	1990_DALYs cases (95% UI)	2021_DALYs cases (95% UI)	Percentage change	1990_ASDR_per 100,000 (95% UI)	2021_ASDR_per 100,000 (95% UI)	EAPC (95% CI)
Andean Latin America	878.65 (372.2–1335.19)	2067.55 (885.21–3191.87)	1.35	4.08 (1.73–6.2)	3.42 (1.47–5.29)	−0.37 (−0.55--0.19)
Australasia	2321.86 (1041.56–3666.34)	2407.45 (1107.74–3958.78)	0.04	10.04 (4.5–15.83)	4.73 (2.13–7.72)	−2.93 (−3.19--2.67)
Caribbean	1409.01 (624.66–2172.41)	1416.24 (646.66–2226.29)	0.01	5.34 (2.36–8.24)	2.66 (1.22–4.18)	−2.31 (−2.59--2.03)
Central Asia	2068.13 (886.15–3138.34)	1569.12 (716.49–2394.64)	−0.24	4.15 (1.76–6.28)	1.83 (0.83–2.8)	−3.63 (−4.13--3.13)
Central Europe	6758.01 (3064.27–10,414)	9227.46 (4266.95–14068.09)	0.37	4.63 (2.09–7.13)	4.3 (1.99–6.54)	−0.56 (−0.93--0.19)
Central Latin America	1585.16 (693.3–2372.86)	4823.62 (2188.24–7620.8)	2.04	1.77 (0.77–2.65)	1.9 (0.87–3.01)	0.47 (0.3–0.64)
Central Sub-Saharan Africa	286.22 (124.14–465.35)	975.71 (403.57–1702.42)	2.41	1.2 (0.52–1.96)	1.61 (0.66–2.85)	1.1 (0.81–1.4)
East Asia	70375.28 (30971.25–115056.56)	52615.66 (20940.64–90261.01)	−0.25	7.3 (3.25–11.78)	2.54 (1.01–4.35)	−3.54 (−3.62--3.45)
Eastern Europe	9279.88 (4286.54–14503.86)	12242.03 (5543.73–18979.54)	0.32	3.38 (1.56–5.29)	3.58 (1.65–5.56)	−1 (−1.62--0.37)
Eastern Sub-Saharan Africa	1130.35 (505.99–1803.82)	1910.27 (844.43–3109.04)	0.69	1.38 (0.63–2.19)	0.99 (0.43–1.6)	−1.46 (−1.6--1.32)
Global	247015.19 (112606.13–380299.24)	305675.95 (135088.79–469863.48)	0.24	6.17 (2.8–9.47)	3.58 (1.58–5.5)	−1.89 (−1.95--1.83)
High-income Asia Pacific	8930.02 (3851.57–14039.19)	22644.81 (10057.56–35310.25)	1.54	4.52 (1.95–7.12)	5.3 (2.37–8.25)	0.85 (0.63–1.07)
High-income North America	30201.78 (13705.36–45783.59)	19658.27 (9128.72–30726.35)	−0.35	8.7 (3.94–13.18)	3.21 (1.5–5.05)	−3.15 (−3.35--2.94)
High-middle SDI	53926.8 (25121.13–84666.67)	61103.1 (26584.05–95508.3)	0.13	5.39 (2.51–8.34)	3.21 (1.39–4.98)	−1.93 (−2.1--1.75)
High SDI	79395.85 (35518.39–120306.05)	81270.66 (36170.94–125964.25)	0.02	7.29 (3.26–11.04)	4.03 (1.8–6.22)	−1.86 (−1.92--1.8)
Low-middle SDI	31283.44 (13697.16–48905.06)	49159.23 (21831.75–74648.11)	0.57	4.61 (2.05–7.16)	3.22 (1.42–4.91)	−1.43 (−1.56--1.29)
Low SDI	4540.77 (2031.54–7088.22)	8741.94 (4066.7–13474.95)	0.93	1.83 (0.82–2.83)	1.53 (0.71–2.35)	−0.9 (−1.15--0.65)
Middle SDI	77615.17 (36217.45–121305.4)	105061.52 (46819.39–162532.37)	0.35	6.75 (3.14–10.53)	3.83 (1.71–5.93)	−1.98 (−2.05--1.9)
North Africa and Middle East	1892.22 (845.93–3064.12)	4636.57 (2100.92–7564.79)	1.45	1.03 (0.46–1.66)	0.92 (0.42–1.51)	−0.54 (−0.65--0.43)
Oceania	13.05 (5.64–21.83)	14.86 (5.73–26.29)	0.14	0.4 (0.17–0.65)	0.18 (0.07–0.32)	−2.53 (−2.84--2.22)
South Asia	20326.82 (9266.55–32394.82)	32,597 (14930.51–49785.39)	0.6	3.13 (1.44–5.01)	2.08 (0.95–3.18)	−1.56 (−1.73--1.38)
Southeast Asia	44395.16 (19963.37–67110.38)	84846.7 (35934.75–129659.38)	0.91	15.82 (7.14–23.9)	12.4 (5.25–18.96)	−0.94 (−1.01--0.87)
Southern Latin America	4323.15 (1968.01–6600.98)	5921.99 (2711.77–9332.27)	0.37	9.47 (4.32–14.44)	6.9 (3.18–10.88)	−0.32 (−0.55--0.08)
Southern Sub-Saharan Africa	263.16 (114.61–409.82)	811.69 (359.77–1290.97)	2.08	0.89 (0.39–1.38)	1.31 (0.58–2.05)	1.48 (1.18–1.79)
Tropical Latin America	3977.59 (1771.11–6054.5)	8760.61 (3971.17–13612.98)	1.2	4.1 (1.82–6.24)	3.36 (1.52–5.23)	−1.07 (−1.28--0.86)
Western Europe	35969.5 (15981.76–54262.43)	35921.74 (15800.34–54483.15)	0	6.31 (2.8–9.5)	3.92 (1.73–5.91)	−1.52 (−1.61--1.43)
Western Sub-Saharan Africa	630.18 (283.85–976.52)	606.61 (259.72–1011.1)	−0.04	0.7 (0.32–1.09)	0.3 (0.13–0.49)	−2.98 (−3.32--2.65)

**Table 2:** Deaths and age-standardized mortality rates (ASMR) of colorectal cancer attributable to low-fiber diet (CRC-LFD) in 1990 and 2021, with percentage change (PC) and estimated annual percentage change (EAPC) from 1990 to 2021.

Location	1990_Death cases (95% UI)	2021_Death cases (95% UI)	Percentage change	1990_ASMR_per 100,000 (95% UI)	2021_ASMR_per 100,000 (95% UI)	EAPC (95% CI)
Andean Latin America	35.81 (15.34–54.81)	88.47 (37.71–137.08)	1.47	0.19 (0.08–0.29)	0.15 (0.07–0.24)	−0.47 (−0.64--0.29)
Australasia	102.19 (45.94–161.93)	118.06 (52.76–195.23)	0.16	0.44 (0.2–0.7)	0.21 (0.09–0.34)	−2.9 (−3.15--2.65)
Caribbean	60.39 (26.5–94.01)	62.45 (27.74–99.33)	0.03	0.24 (0.11–0.38)	0.12 (0.05–0.18)	−2.51 (−2.77--2.25)
Central Asia	70.9 (29.97–107.22)	56.64 (25.36–87.53)	−0.2	0.15 (0.07–0.23)	0.07 (0.03–0.12)	−3.19 (−3.65--2.73)
Central Europe	302.46 (136.15–466.38)	464.09 (212.43–710.73)	0.53	0.21 (0.1–0.33)	0.2 (0.09–0.31)	−0.54 (−0.91--0.17)
Central Latin America	61.41 (26.62–91.83)	196.46 (90.9–309.79)	2.2	0.08 (0.03–0.12)	0.08 (0.04–0.13)	0.22 (0.08–0.37)
Central Sub-Saharan Africa	9.59 (4.18–15.69)	33.02 (13.6–58.62)	2.44	0.05 (0.02–0.08)	0.07 (0.03–0.12)	1.19 (0.88–1.5)
East Asia	2180.29 (960.76–3565.56)	1942.16 (798.95–3317.78)	−0.11	0.27 (0.12–0.44)	0.1 (0.04–0.16)	−3.43 (−3.53--3.32)
Eastern Europe	375.38 (172.6–584.49)	566.93 (261.17–888.72)	0.51	0.14 (0.06–0.22)	0.16 (0.07–0.25)	−0.7 (−1.3--0.09)
Eastern Sub-Saharan Africa	38.44 (17.48–60.75)	65.31 (28.28–105.29)	0.7	0.06 (0.03–0.09)	0.04 (0.02–0.07)	−1.32 (−1.45--1.19)
Global	9689.21 (4409.65–14808.08)	13144.85 (5761.65–20265.1)	0.36	0.27 (0.12–0.42)	0.16 (0.07–0.24)	−1.86 (−1.9--1.82)
High-income Asia Pacific	383.17 (160.6–599.47)	1323.83 (600.7–2055.33)	2.45	0.21 (0.09–0.33)	0.24 (0.11–0.37)	0.78 (0.6–0.96)
High-income North America	1534.23 (703.81–2358.77)	991.51 (448.29–1576.85)	−0.35	0.43 (0.2–0.65)	0.15 (0.07–0.23)	−3.47 (−3.66--3.28)
High-middle SDI	2001.51 (916.59–3117.08)	2665.84 (1154.27–4140.95)	0.33	0.22 (0.1–0.34)	0.14 (0.06–0.21)	−1.63 (−1.82--1.45)
High SDI	3926.85 (1747.28–5985.55)	4449.84 (1995.12–6912.7)	0.13	0.36 (0.16–0.54)	0.19 (0.09–0.3)	−1.97 (−2.05--1.89)
Low-middle SDI	1043.2 (467.15–1621.71)	1784.49 (784.03–2738.33)	0.71	0.18 (0.08–0.28)	0.13 (0.06–0.2)	−1.25 (−1.38--1.11)
Low SDI	152.56 (67.91–236.33)	297.93 (138.37–457.57)	0.95	0.07 (0.03–0.12)	0.06 (0.03–0.1)	−0.74 (−0.96--0.53)
Middle SDI	2554.3 (1181.65–3971.89)	3931.39 (1745.8–6089.88)	0.54	0.26 (0.12–0.41)	0.15 (0.07–0.24)	−1.9 (−1.97--1.84)
North Africa and Middle East	65.65 (29.37–105.73)	162.27 (73.62–266.61)	1.47	0.04 (0.02–0.07)	0.04 (0.02–0.06)	−0.51 (−0.61--0.42)
Oceania	0.41 (0.17–0.68)	0.5 (0.19–0.87)	0.22	0.02 (0.01–0.03)	0.01 (0–0.01)	−2.4 (−2.75--2.05)
South Asia	663.75 (306.61–1067.15)	1166.36 (533.44–1795.38)	0.76	0.12 (0.06–0.2)	0.08 (0.04–0.13)	−1.44 (−1.6--1.28)
Southeast Asia	1524.29 (690.98–2296.46)	3151.93 (1337.57–4821.76)	1.07	0.63 (0.29–0.95)	0.52 (0.22–0.8)	−0.8 (−0.86--0.73)
Southern Latin America	198.47 (89.24–299.37)	284.39 (127.3–446.1)	0.43	0.46 (0.21–0.69)	0.32 (0.14–0.5)	−0.48 (−0.71--0.25)
Southern Sub-Saharan Africa	9.72 (4.27–14.79)	29.62 (13.22–46.3)	2.05	0.04 (0.02–0.06)	0.06 (0.02–0.09)	1.23 (0.97–1.49)
Tropical Latin America	148.11 (66.05–224.9)	338.1 (152.71–528.64)	1.28	0.18 (0.08–0.27)	0.13 (0.06–0.21)	−1.32 (−1.55--1.1)
Western Europe	1899.83 (837.74–2902.12)	2079.42 (918.65–3167.87)	0.09	0.32 (0.14–0.48)	0.19 (0.09–0.29)	−1.58 (−1.68--1.49)
Western Sub-Saharan Africa	24.74 (11.17–38.29)	23.32 (10.01–38.63)	−0.06	0.03 (0.01–0.05)	0.01 (0.01–0.02)	−2.8 (−3.1--2.51)

**Figure 1:**
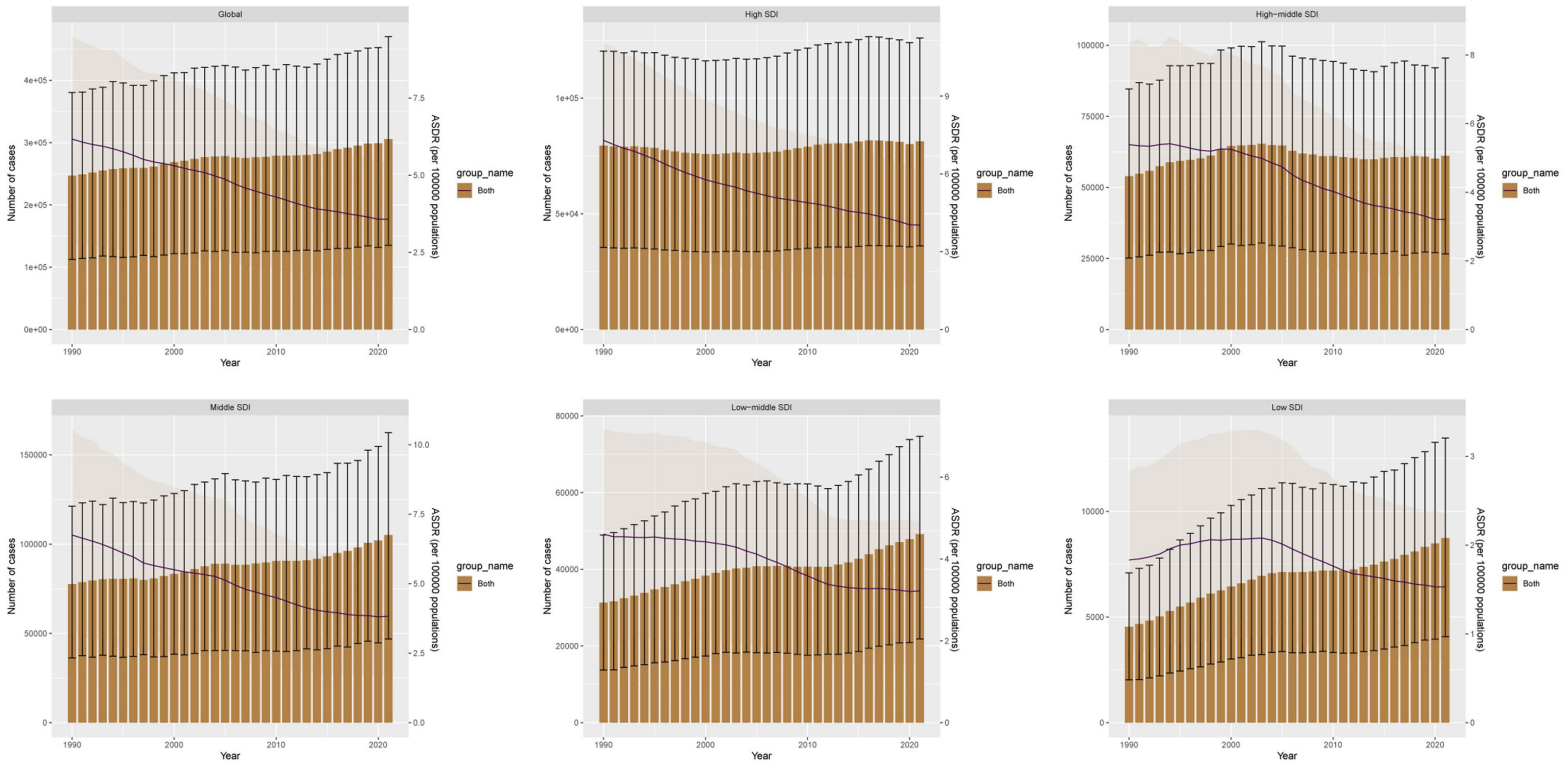
Disability-adjusted life years (DALYs) cases and age-standardized DALY rate (ASDR) of colorectal cancer attributable to low-fiber diet (CRC-LFD) from 1990 to 2021.

Across SDI regions, the middle SDI region recorded the highest number of DALYs in 2021 [105,061.52 (95% UI: 46,819.39 to 162,532.37)], reflecting a 35% increase since 1990. In contrast, the high SDI region had the highest ASDR [4.03 (95% UI: 1.80 to 6.22) per 100,000], with an EAPC of −1.86 (95% CI: −1.92 to −1.80). This region also reported the highest number of deaths [4,449.84 (95% UI: 1,995.12 to 6,912.70)], representing a 13% increase, and the highest ASMR [0.19 (95% UI: 0.09 to 0.30) per 100,000], with an EAPC of −1.97 (95% CI: −2.05 to −1.89) (Figure 1). These findings indicate that the absolute burden was greatest in middle SDI settings, likely because of large population size and ongoing demographic transition, whereas the standardized burden remained concentrated in high SDI regions, where CRC incidence, life expectancy, and diet-related risk exposure are relatively high.

Among the 21 GBD regions, Central Sub-Saharan Africa experienced the most pronounced increase in DALYs (241%), whereas High-income Asia Pacific showed the largest rise in deaths (245%). Southern Latin America had the highest ASDR [6.90 (95% UI: 3.18 to 10.88) per 100,000], while Southeast Asia exhibited the highest ASMR [0.52 (95% UI: 0.22 to 0.80) per 100,000]. Notably, Southern Sub-Saharan Africa demonstrated the fastest increases in ASDR and ASMR, with EAPCs of 1.48 (95% CI: 1.18 to 1.79) and 1.23 (95% CI: 0.97 to 1.49), respectively (Supplementary Figure 1). These regional patterns suggest substantial heterogeneity in CRC-LFD burden. In particular, the increase in age-standardized rates in Southern Sub-Saharan Africa may indicate a true rise in age-specific burden rather than a change driven only by population growth, highlighting the need for targeted surveillance in emerging high-risk regions.

### National burden of CRC-LFD

**Figure 2:**
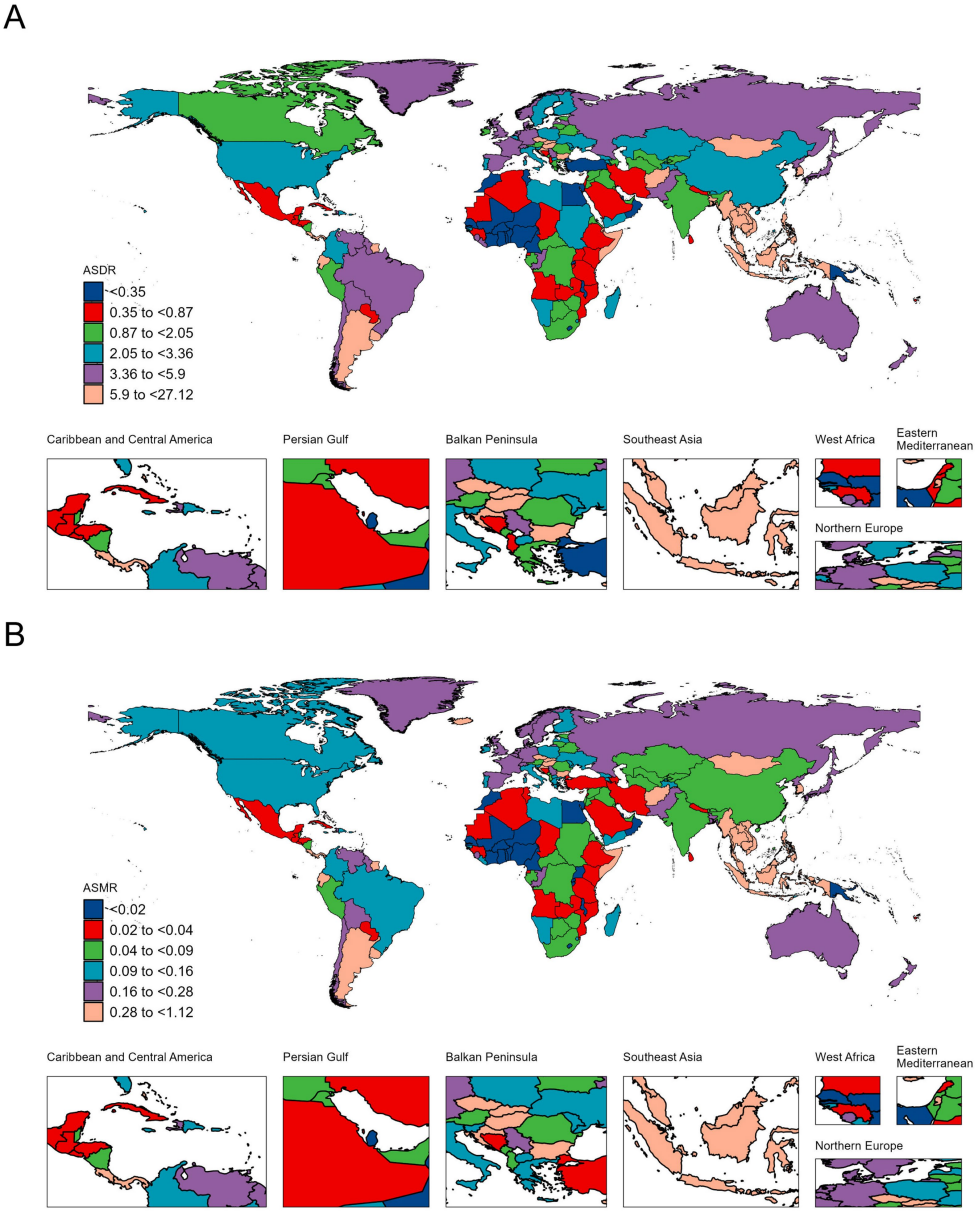
Country-level distribution of age-standardized DALY rate (ASDR) and age-standardized mortality rate (ASMR) of colorectal cancer attributable to low-fiber diet (CRC-LFD) per 100,000 population in 2021: **(A)** ASDR of CRC-LFD across 204 countries and territories in 2021; **(B)** ASMR of CRC-LFD across 204 countries and territories in 2021.

Over the past 32 years, the top three countries with the fastest increases in DALYs were Burundi, Iraq, and the Democratic Republic of the Congo, with percentage changes of 958, 788, and 751%, respectively (Figure 2A). Similarly, the top three countries with the fastest growth in deaths were Burundi, Iraq, and the Democratic Republic of the Congo, with percentage changes of 857, 766, and 745%, respectively (Figure 2B). In terms of ASDR, the three countries with the most rapid increases were Burundi, Democratic Republic of the Congo, and Iraq, with EAPCs of 4.68 (95% CI: 3.92 to 5.44), 4.25 (95% CI: 3.37 to 5.14), and 2.98 (95% CI: 2.17 to 3.81), respectively. For ASMR, the fastest-growing countries were also Burundi, Democratic Republic of the Congo, and Iraq, with EAPCs of 4.42 (95% CI: 3.73 to 5.12), 4.16 (95% CI: 3.30 to 5.03), and 3.09 (95% CI: 2.33 to 3.85), respectively (Supplementary Tables 1, 2). The simultaneous increase in both absolute numbers and age-standardized rates suggests that these countries may represent emerging CRC-LFD hotspots, where demographic change, dietary transition, limited screening coverage, and constrained health-system capacity may jointly contribute to the rapid rise in burden.

### Age and sex differences in the burden of CRC-LFD

**Figure 3:**
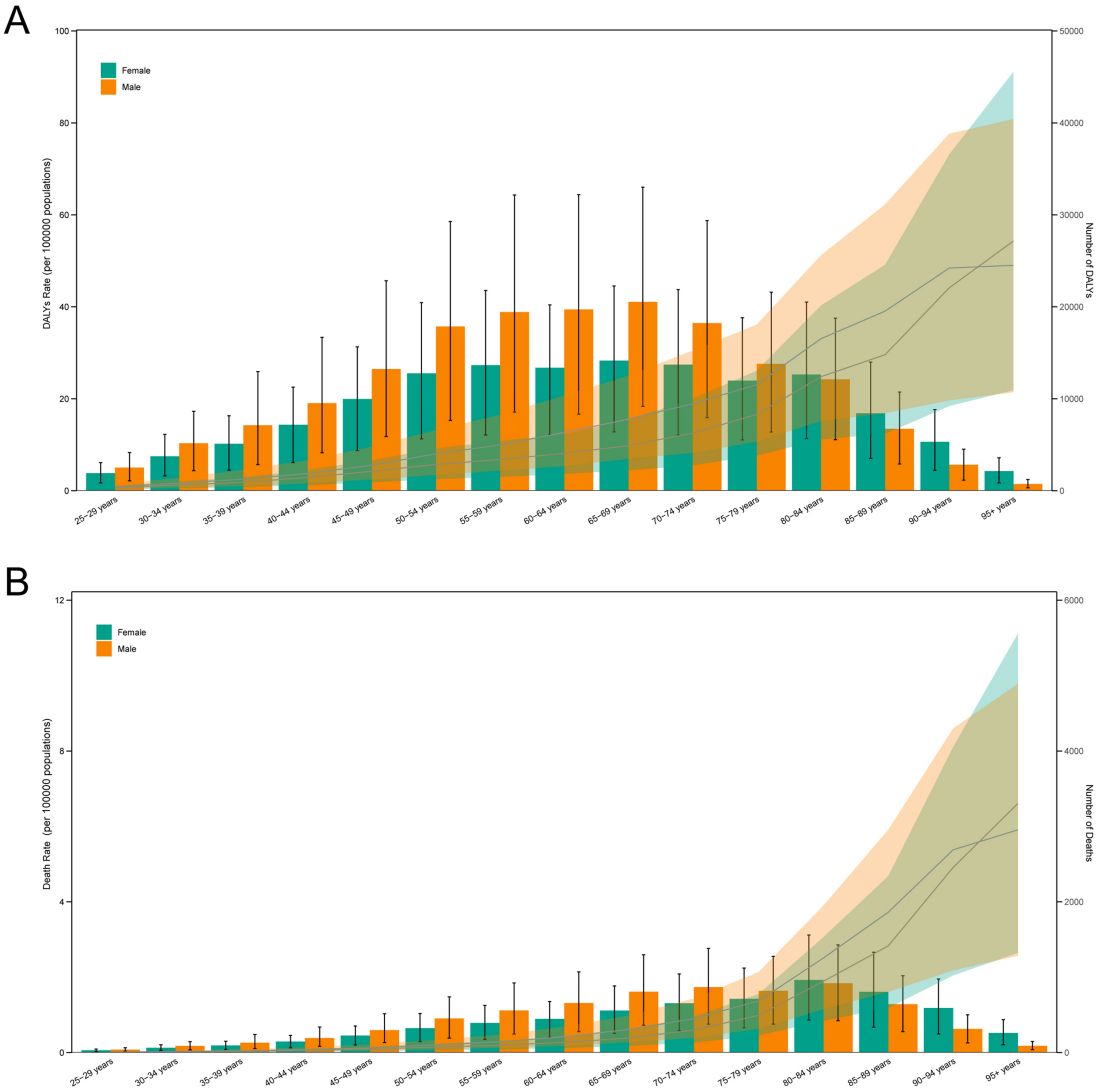
Age-specific numbers and rates of disability-adjusted life years (DALYs) **(A)** and deaths **(B)** of colorectal cancer attributable to low-fiber diet (CRC-LFD) by age and sex in 2021.

The DALYs of CRC-LFD were highest in the 65–69 age group for both males and females, with 20,541 and 14,149 cases, respectively (Figure 3A). Deaths peaked in the 80–84 age group for both males and females, with 920 and 964 cases, respectively (Figure 3B). However, males under 80 years had significantly higher DALYs than females, whereas females over 80 years had higher DALYs than males. ASDR and ASMR increased with age. Among individuals under 95 years old, males had significantly higher ASDR and ASMR than females. These findings indicate that CRC-LFD is predominantly an aging-related burden, with deaths shifting toward the oldest age groups. The higher burden among males before very old age may reflect sex differences in CRC risk, dietary behavior, metabolic risk profiles, and screening participation, while the higher absolute burden among females over 80 may be partly explained by longer female life expectancy and larger numbers of women surviving into advanced age.

### The association between CRC-LFD burden and SDI

**Figure 4:**
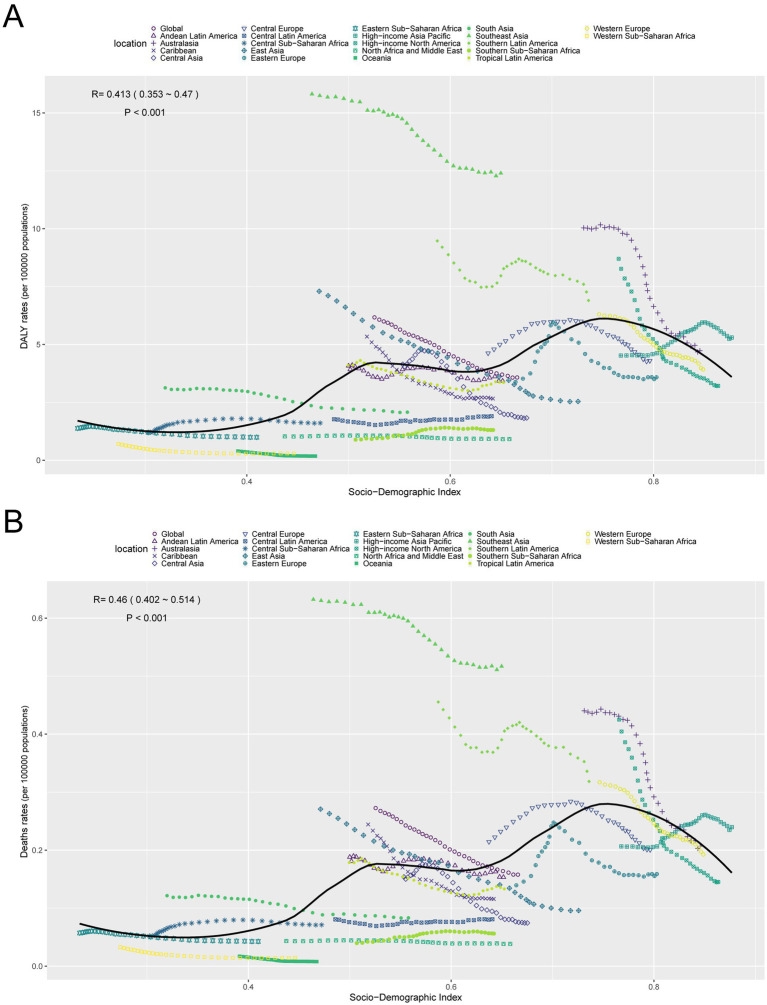
Age-standardized DALY rate (ASDR) **(A)** and age-standardized mortality rate (ASMR) **(B)** of colorectal cancer attributable to low-fiber diet (CRC-LFD) in 21 Global Burden of Disease (GBD) regions by Socio-demographic Index (SDI), 1990–2021.

In 2021, ASDR and ASMR of CRC-LFD showed a significant positive correlation with SDI, indicating that the overall disease burden increased with socioeconomic development (Figures 4A,B). Specifically, the burden showed a marked increase when SDI ranged from 0.4 to 0.5, remained relatively stable between 0.5 and 0.6, then rose again and peaked at an SDI of 0.6–0.75. When SDI exceeded 0.75, the burden gradually declined. This nonlinear relationship suggests that CRC-LFD burden may increase during socioeconomic transition, when dietary westernization, reduced fiber intake, urbanization, and improved cancer detection may occur simultaneously. The subsequent decline at very high SDI levels may reflect better screening, earlier diagnosis, improved treatment, and greater health awareness.

Notably, some regions—including Southeast Asia and Southern Latin America—had CRC-LFD burdens significantly higher than expected, whereas North Africa and the Middle East, Southern Sub-Saharan Africa, Western Sub-Saharan Africa, and Central Latin America showed burdens markedly lower than projected. These deviations indicate that SDI alone cannot fully explain CRC-LFD burden. Regional dietary structures, intake of whole grains, fruits, vegetables and legumes, CRC screening coverage, registry completeness, obesity prevalence, and competing mortality risks may all influence the observed patterns.

### Decomposition analysis of CRC-LFD burden

**Figure 5:**
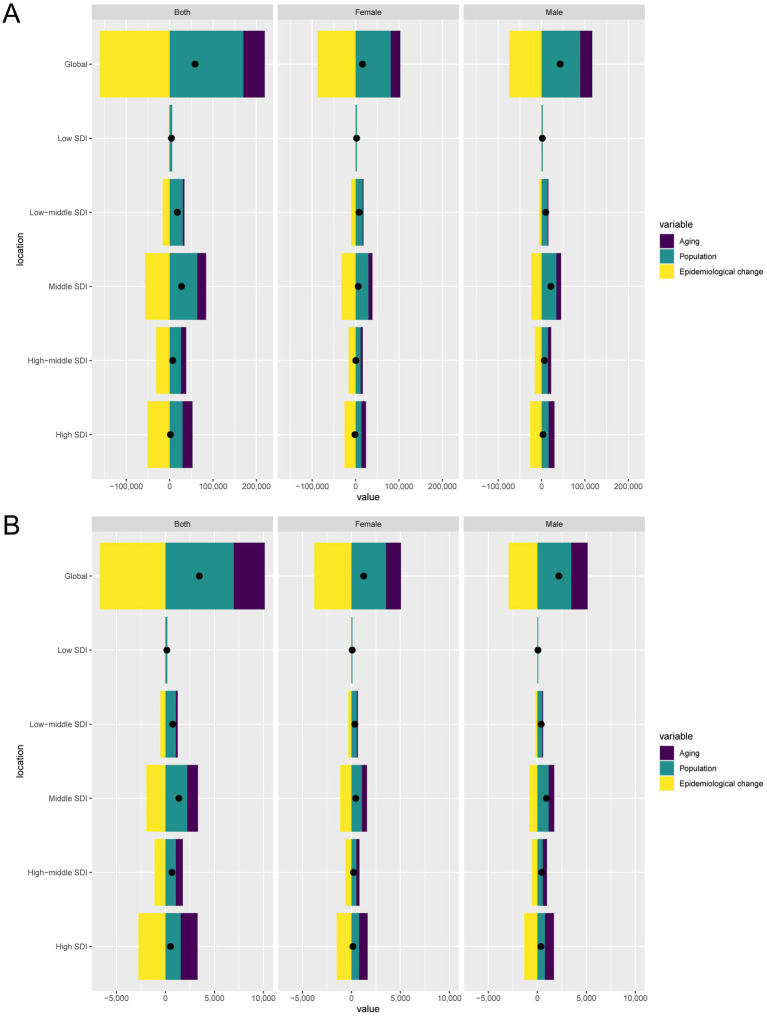
Decomposition analysis of changes in disability-adjusted life years (DALYs) **(A)** and deaths **(B)** of colorectal cancer attributable to low-fiber diet (CRC-LFD) between 1990 and 2021 across Socio-demographic Index (SDI) regions.

Decomposition analysis was conducted to quantify the contributions of population growth, aging, and epidemiological changes to the burden of CRC-LFD. From 1990 to 2021, global DALYs increased by 58,660.75. Population growth contributed 169,787.78 cases (289.44%), aging contributed 49,335.04 cases (84.1%), while epidemiological changes resulted in a reduction of 160,462.07 cases (−273.54%) (Figure 5A).

For deaths, the global increase was 3,455.64. Population growth contributed 6,981.21 cases (202.02%), aging contributed 3,147.89 cases (91.09%), and epidemiological changes reduced deaths by 6,673.46 cases (−193.12%). The middle SDI region showed the greatest increases in DALYs (20,350.47) and deaths (1,377.09). Specifically, population growth contributed 63,696.15 DALYs (232.08%) and 2,234.35 deaths (162.25%), aging contributed 20,350.47 DALYs (74.15%) and 1,077.97 deaths (78.28%), while epidemiological changes led to reductions of 56,600.27 DALYs (−206.22%) and 1,935.24 deaths (−140.53%) (Figure 5B). These findings explain the apparent contradiction between rising absolute burden and declining age-standardized rates: although age-specific CRC-LFD burden has decreased, population growth and aging have outweighed these improvements, resulting in continued increases in DALYs and deaths.

### Cross-country inequality analysis of CRC-LFD burden

**Figure 6:**
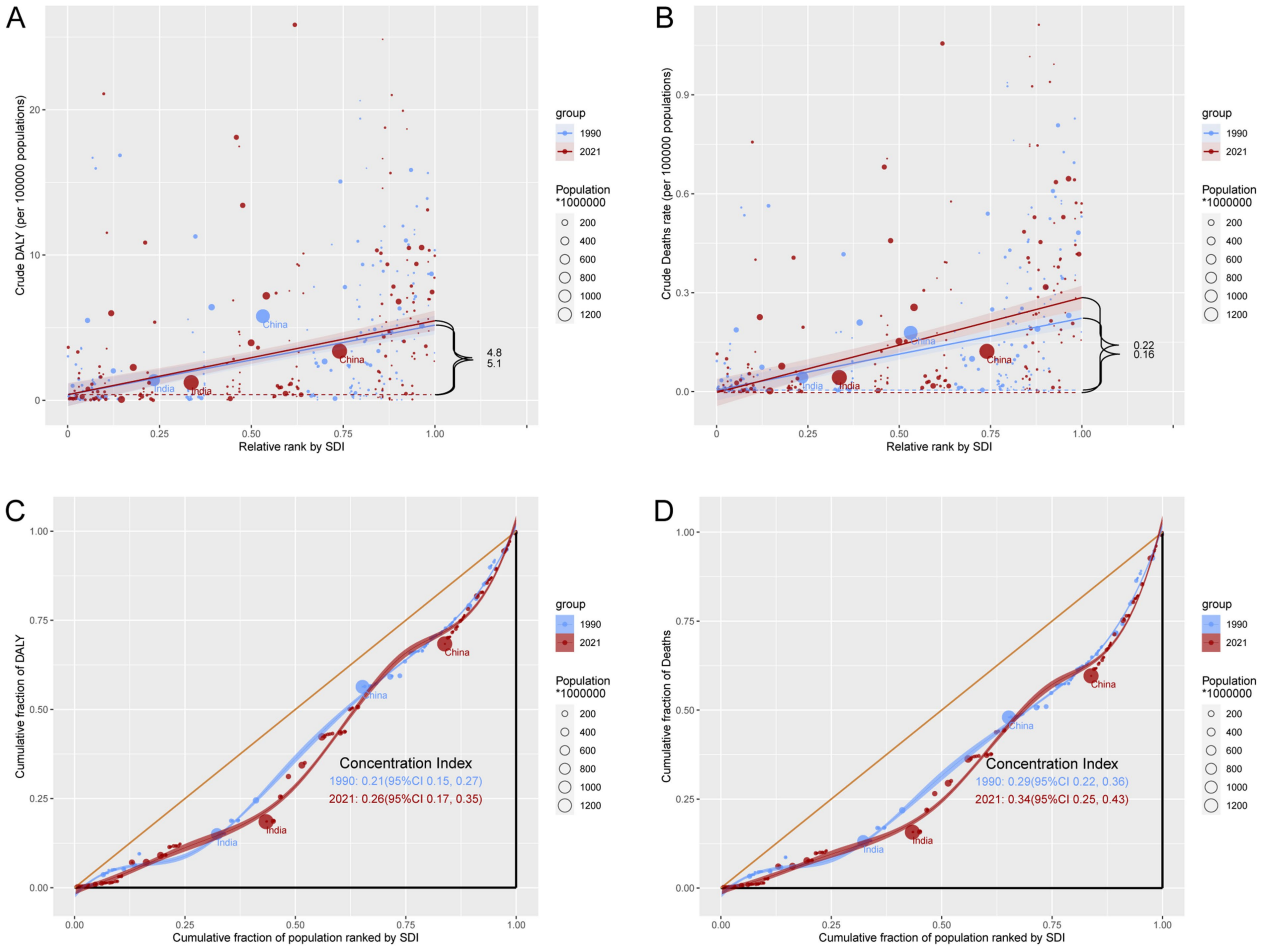
Inequality analysis of disability-adjusted life years (DALYs) and mortality of colorectal cancer attributable to low-fiber diet (CRC-LFD) in 1990 and 2021 worldwide. **(A)** Health inequality regression curves for DALYs. **(B)** Health inequality regression curves for mortality. **(C)** Concentration curves for DALYs. **(D)** Concentration curves for mortality.

Over the past 32 years, the SII did not change substantially. Specifically, the SII of DALY rates increased slightly from 4.8 in 1990 to 5.1 in 2021, while the SII of death rates increased from 0.16 to 0.22, indicating a widening absolute disparity between the highest and lowest SDI regions (Figures 6A,B). The CI of DALY rates increased from 0.21 in 1990 to 0.26 in 2021, while the CI of death rates increased from 0.29 to 0.34 (Figures 6C,D). These results suggest that CRC-LFD burden has become increasingly concentrated in higher-SDI regions, and that relative inequality has worsened over time.

This pattern differs from some health burdens that are concentrated in low SDI settings because of undernutrition or infection-related mechanisms. LFD, as a dietary risk factor for CRC, appears more closely linked to socioeconomic development, urbanization, westernized dietary patterns, and aging population structures. Therefore, reducing CRC-LFD burden requires not only improving food availability but also promoting healthier dietary composition, particularly higher intake of fiber-rich foods such as whole grains, legumes, fruits, and vegetables.

### Prediction analysis of CRC-LFD burden

**Figure 7:**
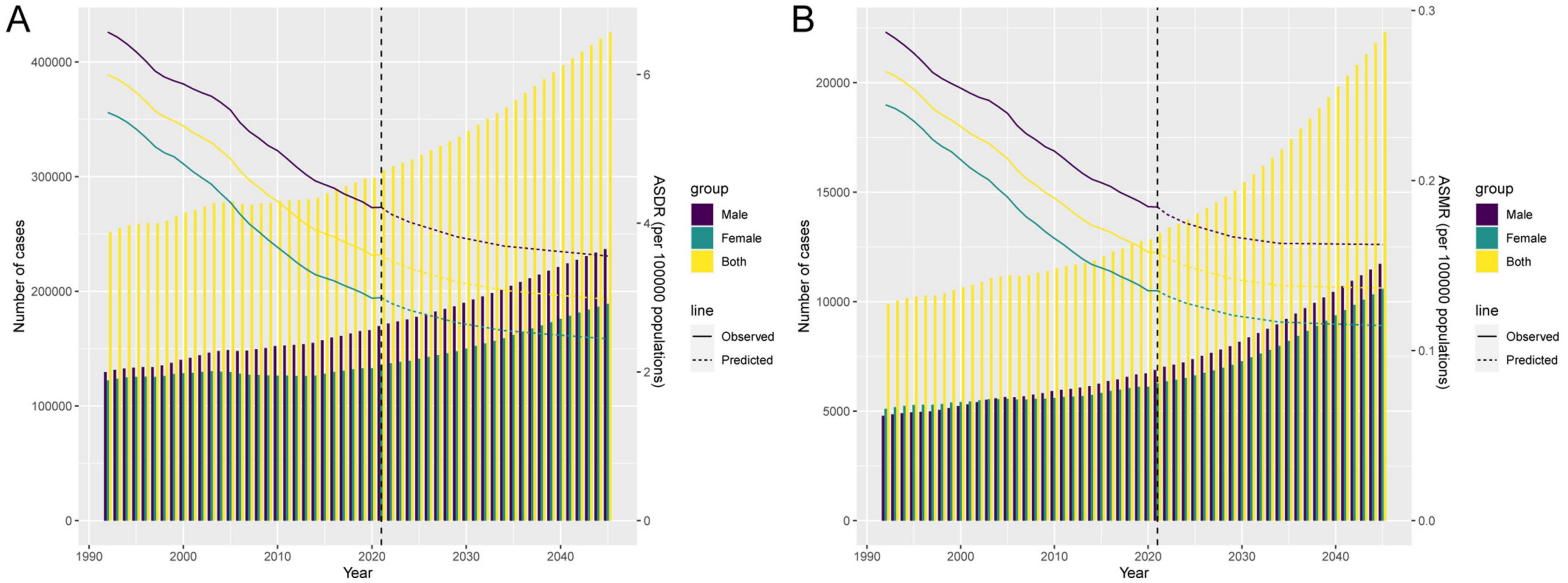
Projections of the temporal trends in disability-adjusted life years (DALYs) cases, death cases, age-standardized DALY rate (ASDR), and age-standardized mortality rate (ASMR) of colorectal cancer attributable to low-fiber diet (CRC-LFD) globally up to 2045. **(A)** The number and ASDR of CRC-LFD by year and sex. **(B)** The number and ASMR of CRC-LFD by year and sex.

From 2022 to 2045, the burden of CRC-LFD is projected to continue rising in terms of DALYs and deaths (Figures 7A,B). By 2045, DALYs are expected to reach 426,001, and deaths are projected to reach 22,313, with males consistently experiencing a higher burden than females. However, ASDR and ASMR are expected to continue declining. By 2045, ASDR is projected to decline to 2.98 per 100,000, and ASMR to 0.14 per 100,000. This projected divergence indicates that demographic forces, particularly population aging, will remain the main driver of future absolute burden even if age-standardized rates continue to improve.

## Discussion

This study systematically assessed the global, regional, and national burden of CRC-LFD from 1990 to 2021. The findings revealed a seemingly paradoxical pattern: although ASDR and ASMR showed overall declines, the absolute numbers of DALYs and deaths continued to increase. This pattern suggests that improvements in age-specific risk, diagnosis, or treatment have not been sufficient to offset the effects of population growth and aging. Therefore, CRC-LFD should not be interpreted merely as a declining age-standardized risk, but rather as a persistent and demographically amplified public health burden.

The increase in absolute burden is closely linked to global nutritional transition. Over recent decades, many countries have experienced rapid shifts from traditional diets rich in whole grains, legumes, vegetables, and other fiber-containing foods toward more Westernized dietary patterns characterized by refined carbohydrates, processed foods, red and processed meat, high fat intake, and lower dietary fiber density (31). This transition has been accelerated by urbanization, changes in food supply chains, increased consumption of ultra-processed foods, and reduced reliance on locally produced plant-based foods (32). In this context, low-fiber intake rarely occurs as an isolated dietary exposure; instead, it often clusters with broader unhealthy dietary patterns, physical inactivity, obesity, metabolic dysfunction, and other lifestyle-related risks (33). Therefore, the burden of CRC-LFD should be interpreted within the broader context of nutrition-related disease transition, rather than merely regarded as the independent effect of a single dietary component.

The biological plausibility of the observed epidemiological patterns is supported by several mechanisms. Dietary fiber increases stool bulk, shortens intestinal transit time, dilutes carcinogens in the colonic lumen, and promotes the production of short-chain fatty acids, particularly butyrate, through microbial fermentation (10). Butyrate has anti-inflammatory and anti-proliferative effects and may contribute to the maintenance of intestinal epithelial integrity (34, 35). Conversely, insufficient fiber intake may alter the gut microbiota, reduce beneficial metabolites, promote chronic low-grade inflammation, and increase exposure of the colorectal mucosa to bile acids and other potentially carcinogenic compounds (36). These mechanisms are consistent with the higher CRC-LFD burden observed in populations undergoing dietary Westernization and rising obesity prevalence (37). Obesity may further amplify CRC risk through insulin resistance, chronic inflammation, adipokine dysregulation, and metabolic disturbances, thereby interacting with low-fiber dietary patterns in shaping long-term CRC burden (38).

Substantial heterogeneity across SDI regions indicates that socioeconomic development modifies both exposure patterns and disease outcomes. In high SDI regions, historically high consumption of processed and Westernized diets may partly explain the concentration of CRC-LFD burden. However, declining age-standardized rates in some high-income settings may reflect better access to screening, earlier diagnosis, improved treatment, and stronger public health awareness. By contrast, middle SDI regions may face a dual challenge: rapid dietary transition and urbanization increase exposure to CRC-related dietary risks, while screening systems and cancer care infrastructure may not yet be fully developed. This may explain why middle SDI regions contributed substantially to the absolute burden. In low SDI settings, the currently lower recorded burden may partly reflect younger population structures, underdiagnosis, incomplete cancer registration, and limited access to diagnostic services rather than genuinely low disease risk. Therefore, differences in CRC-LFD burden across SDI strata should be interpreted through both dietary exposure and health-system capacity (39).

The rapid increase in CRC-LFD burden in parts of Sub-Saharan Africa and other developing regions deserves particular attention. These regions are undergoing demographic expansion, urbanization, and gradual changes in dietary patterns, while cancer screening and treatment systems remain limited (40, 41). Increases in observed DALYs and deaths may partly reflect improved case detection and data availability, but they also suggest an emerging shift in non-communicable disease burden. If dietary Westernization continues without parallel improvements in prevention, screening, and treatment, these regions may experience a delayed but substantial rise in CRC burden. In contrast, high-income Asia Pacific showed increasing deaths despite relatively advanced healthcare systems, which may be strongly influenced by rapid population aging (42). This highlights that even regions with effective healthcare infrastructure cannot fully avoid increasing absolute burden when demographic aging is pronounced.

Sex- and age-specific patterns further support the need for targeted interpretation. The higher burden among males before very old age may reflect higher exposure to behavioral risk factors, including lower adherence to healthy dietary patterns, greater consumption of red and processed meat, smoking, alcohol use, and higher visceral adiposity (43). Biological factors, such as sex hormone profiles, immune regulation, and differences in gut microbiota composition, may also contribute to sex disparities in CRC risk (6). Among older age groups, particularly after 80 years, the burden among females may become relatively higher because of longer female life expectancy and greater accumulation of elderly women in the population (7). Thus, sex differences in CRC-LFD burden likely reflect the combined effects of behavior, biology, and population structure.

The decomposition analysis provides an important explanation for the divergence between declining age-standardized rates and rising absolute burden. Population growth and aging were major forces driving increases in DALYs and deaths, whereas epidemiological changes partly offset these increases. This suggests that improvements in prevention, diagnosis, or treatment may have reduced age-specific burden, but demographic pressure has continued to expand the total number of affected individuals. This finding is particularly important for public health planning: a decline in ASDR or ASMR does not necessarily mean that health-system demand will decrease. On the contrary, countries with rapidly aging populations may still face increasing numbers of CRC cases, deaths, and disability even under improving age-standardized trends (43).

The inequality analysis also indicates that CRC-LFD burden is unevenly distributed across countries and regions. A greater concentration of burden in higher-SDI settings may reflect historically higher exposure to Westernized diets and better ascertainment of cancer outcomes (44). However, the emerging increases in several lower-SDI and middle-SDI countries suggest that global disparities may evolve over time. Countries at earlier stages of nutritional transition may experience future increases if preventive strategies are not implemented early. Therefore, CRC-LFD prevention should not be limited to high-income settings; it should also be integrated into non-communicable disease prevention frameworks in low- and middle-income countries before the burden becomes entrenched.

Compared with previous GBD-based studies on CRC and dietary risk factors, our findings are generally consistent in showing that dietary risks remain important contributors to CRC burden and that absolute burden continues to rise despite improvements in age-standardized indicators. Similar patterns have been reported for other diet-related risks, such as diets high in red meat, high in processed meat, low in whole grains, and low in calcium (43). However, the burden attributable to LFD has distinct implications because dietary fiber intake is closely tied to broader food systems, affordability of healthy foods, cultural dietary traditions, and public nutrition policy. Unlike some risk factors that can be addressed primarily through reduction strategies, improving fiber intake requires both increasing access to fiber-rich foods and reshaping long-term dietary behavior (8). Therefore, CRC-LFD should be viewed as a modifiable but structurally embedded risk factor.

### Public health implications

These findings highlight the need to incorporate dietary fiber promotion into CRC prevention strategies. Public health policies should encourage the consumption of whole grains, legumes, fruits, and vegetables while reducing reliance on refined grains and ultra-processed foods. Population-level measures, including nutrition education, front-of-package labeling, healthy school and workplace meals, and improved affordability of fiber-rich foods, may help increase fiber intake (45).

Prevention strategies should also be tailored to socioeconomic context. High SDI regions should focus on sustaining healthy dietary habits and integrating dietary counseling into CRC screening programs. Middle SDI regions require early intervention during nutritional transition and rapid urbanization. Low SDI regions should prioritize dietary surveillance, cancer registration, and basic screening capacity. Given the rising absolute burden driven by aging and population growth, dietary intervention should be combined with improved screening, early diagnosis, and equitable access to cancer care. Overall, reducing CRC-LFD burden requires coordinated action across nutrition policy, healthcare systems, and broader food environments.

### Limitations

This study has several limitations. First, GBD 2021 estimates are modelled rather than directly observed data. In countries with weak cancer registration, mortality surveillance, or dietary monitoring systems, especially low- and middle-income countries, estimates may rely more heavily on statistical modelling and data borrowing, leading to potential uncertainty and bias. Second, dietary fiber exposure may be affected by limitations of dietary surveys, including recall bias, underreporting, inconsistent assessment tools, and cultural differences in food composition. Within-country differences in fiber intake by age, sex, socioeconomic status, and urbanization may also not be fully captured. Third, this ecological study used aggregated population-level data, so ecological bias cannot be excluded. The findings should not be interpreted as individual-level causal relationships. Low-fiber intake often coexists with other unhealthy behaviors, such as high red and processed meat intake, obesity, physical inactivity, smoking, and alcohol use, which may cause residual confounding. Fourth, although GBD uses comparative risk assessment methods, this study cannot establish causality between LFD and CRC burden. In addition, no sensitivity analysis was conducted to test the robustness of results under alternative exposure definitions or modelling assumptions. Overall, these limitations suggest that our findings should be interpreted as population-level estimates of attributable burden rather than direct causal evidence.

## Conclusion

From 1990 to 2021, the global burden of CRC-LFD showed a clear divergence between absolute burden and age-standardized rates. Although ASDR and ASMR declined, the numbers of DALYs and deaths continued to increase, indicating that population growth and aging were the principal drivers of the expanding absolute burden. This finding suggests that improvements in age-specific risk or outcomes have not been sufficient to offset demographic pressure. Substantial socioeconomic and geographic disparities were observed. The burden remained concentrated in higher-SDI settings in terms of age-standardized rates, while several low- and middle-SDI countries experienced rapid increases, reflecting the combined influence of dietary transition, uneven health-resource allocation, and differences in cancer prevention and care capacity. Therefore, prevention strategies should not only promote adequate dietary fiber intake and healthy dietary patterns, but also address inequalities in health education, screening accessibility, early diagnosis, and treatment availability.

Future research should move beyond ecological analyses based on modeled estimates. Individual-level prospective cohort studies are needed to clarify the causal relationship between dietary fiber intake and CRC outcomes across different populations. Intervention trials are also warranted to evaluate whether fiber-promoting dietary strategies can reduce CRC burden. In addition, region-specific analyses, particularly in countries with rapidly increasing burden, may help identify locally relevant dietary, healthcare, and socioeconomic determinants and support more targeted public health policies.

## Data Availability

The original contributions presented in the study are included in the article/Supplementary material, further inquiries can be directed to the corresponding author.
