# Supplementary figures and images for: Global, regional and national burden of colorectal cancer attributable to low-fiber diet from 1990 to 2021: a systematic analysis of the global burden of disease study 2021
Source: Front Nutr. 2026 Jun 4;13:1688108. doi: 10.3389/fnut.2026.1688108 (PMC13275277; doi:10.3389/fnut.2026.1688108)

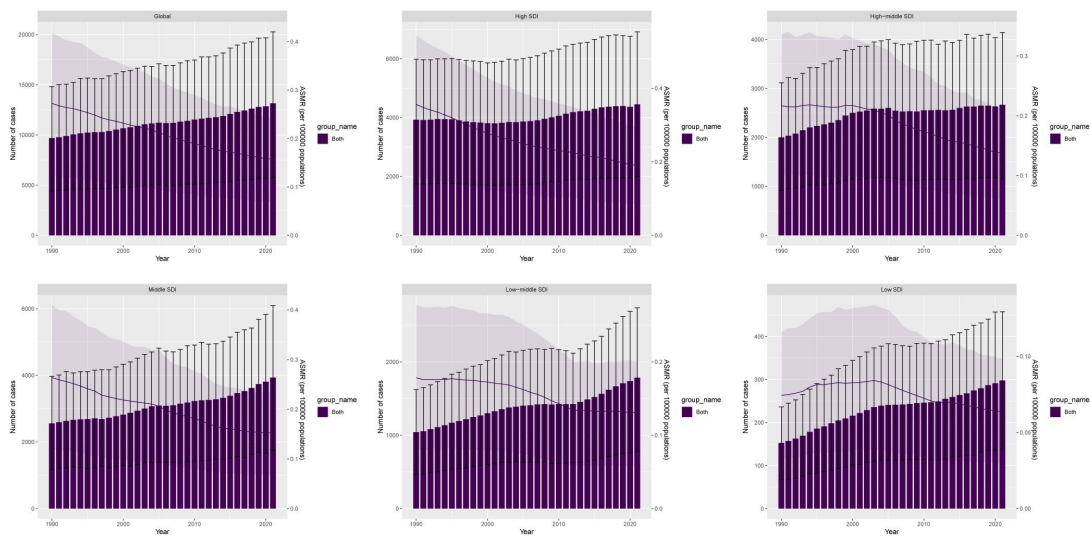

Supplementary figure 1. Death cases and ASMR of CRC-LFD from 1990 to 2021.

Supplement: Supplementary file 1 [file Image_1.PDF]
