# Supplementary material for: Global, regional and national burden of colorectal cancer attributable to low-fiber diet from 1990 to 2021: a systematic analysis of the global burden of disease study 2021
Source: Front Nutr. 2026 Jun 4;13:1688108. doi: 10.3389/fnut.2026.1688108 (PMC13275277; doi:10.3389/fnut.2026.1688108)
Supplement: Supplementary file 2 [file Table_1.PDF]

Location	1990_DALYs cases (95% UI)	2021_DALYs cases (95% UI)	Percentage change	1990_ASDR_per 100000(95% UI)	2021_ASDR_per 100 000(95% UI)	EAPC (95% CI)
Afghanistan	244.54 (66.99-483.21)	1037.55 (344.32-2004.16)	3.24	2.46 (0.67-4.86)	3.32 (1.1-6.42)	1.77 (0.89-2.67)
Albania	39.38 (17.12-62.62)	36.04 (14.15-62.05)	-0.08	1.19 (0.52-1.89)	1.35 (0.53-2.33)	-3.65 (-4.12--3.18)
Algeria	152.66 (70.19-237.14)	123.02 (49.11-210.34)	-0.19	0.6 (0.28-0.94)	0.28 (0.11-0.48)	-4.8 (-5.3--4.3)
American Samoa	0.13 (0.06-0.23)	0.1 (0.03-0.2)	-0.23	0.28 (0.12-0.47)	0.2 (0.06-0.4)	-2.08 (-2.42--1.73)
Andorra	2.28 (0.8-4.1)	3.95 (1.46-7.21)	0.73	4.19 (1.48-7.55)	4.62 (1.71-8.43)	-1.18 (-1.55--0.81)
Angola	126.71 (53.84-213.86)	90.85 (37.78-159.07)	-0.28	1.23 (0.52-2.08)	0.28 (0.12-0.49)	-5.86 (-6.45--5.27)
Antigua and Barbuda	9.62 (4.23-14.62)	14.87 (6.85-22.75)	0.55	15.98 (7.02-24.28)	16.64 (7.67-25.45)	-0.94 (-1.09--0.79)
Argentina	3094.33 (1429.32-4750.29)	4693.49 (2131.71-7440.98)	0.52	9.35 (4.32-14.35)	10.32 (4.69-16.36)	0.43 (0.12-0.75)
Armenia	145.79 (65.21-223.59)	99.38 (42.7-157.66)	-0.32	4.26 (1.91-6.54)	3.32 (1.43-5.26)	-3.75 (-4.25--3.24)
Australia	2022.27 (903.38-3163.76)	2026.7 (925.94-3351.86)	0	12 (5.36-18.77)	7.86 (3.59-13)	-3.21 (-3.53--2.88)
Austria	624.39 (286.95-958.51)	291.1 (130.57-496.28)	-0.53	8.04 (3.69-12.34)	3.24 (1.45-5.53)	-3.87 (-4.13--3.61)
Azerbaijan	190.53 (88.55-300.51)	97.58 (37.79-163.74)	-0.49	2.6 (1.21-4.1)	0.93 (0.36-1.56)	-6.19 (-6.87--5.51)
Bahrain	1.49 (0.62-2.77)	4.33 (1.48-8.14)	1.91	0.29 (0.12-0.55)	0.28 (0.1-0.53)	-1.99 (-2.18--1.8)
Bangladesh	5993.3	9865.94	0.65	5.49	5.99	-1.81

	(2703.56-9596.97)	(4415.92-16430.2)		(2.48-8.79)	(2.68-9.98)	(-1.92--1.7)
Barbados	27.79 (11.91-43.27)	43.64 (17.66-71.82)	0.57	10.97 (4.7-17.07)	14.59 (5.91-24.02)	0.18 (-0.06-0.41)
Belarus	73.71 (29.53-134.3)	136.3 (55.81-255.57)	0.85	0.71 (0.28-1.29)	1.46 (0.6-2.74)	0.23 (-0.66-1.13)
Belgium	1069.32 (481.04-1612.36)	773.25 (346.8-1256.85)	-0.28	10.72 (4.82-16.16)	6.74 (3.02-10.96)	-2.24 (-2.37--2.1)
Belize	1.26 (0.57-1.94)	4.01 (1.77-6.53)	2.18	0.67 (0.3-1.04)	0.94 (0.41-1.52)	0 (-0.32-0.33)
Benin	2.34 (0.92-4.34)	4.59 (1.72-9.18)	0.96	0.05 (0.02-0.09)	0.03 (0.01-0.07)	-0.32 (-0.99-0.34)
Bermuda	3.29 (1.43-5.16)	11.87 (4.98-19.28)	2.61	5.54 (2.41-8.69)	18.67 (7.83-30.35)	2.04 (1.59-2.49)
Bhutan	13.68 (5.74-23.19)	13.55 (5.55-24.14)	-0.01	2.17 (0.91-3.68)	1.79 (0.73-3.19)	-2.84 (-2.96--2.73)
Bolivia	132.05 (54.78-216.99)	376.52 (153.68-652.4)	1.85	2.07 (0.86-3.4)	3.19 (1.3-5.53)	-0.02 (-0.28-0.25)
Bosnia and Herzegovina	18.49 (7.35-32.96)	25.03 (9.32-49.27)	0.35	0.41 (0.16-0.73)	0.76 (0.28-1.49)	-0.38 (-0.85-0.1)
Botswana	10.45 (4.57-17.15)	26.63 (11.16-46.67)	1.55	0.79 (0.35-1.3)	1.11 (0.47-1.95)	-0.51 (-1.06-0.04)
Brazil	3965.9 (1765.3-6037.03)	8718.57 (3950.1-13562.58)	1.2	2.67 (1.19-4.07)	3.96 (1.79-6.15)	-1.09 (-1.3--0.87)
Brunei	10.2 (4.51-16.28)	45.05 (19.6-73.11)	3.42	3.93 (1.74-6.28)	9.99 (4.34-16.2)	2.25 (1.9-2.6)
Bulgaria	806 (362.25-1258.19)	1273.47 (541.61-2112.69)	0.58	9.29 (4.17-14.5)	18.76 (7.98-31.13)	0.66 (-0.06-1.38)
Burkina Faso	13.77 (5.69-23.81)	17.22 (6.35-31.71)	0.25	0.14 (0.06-0.25)	0.08 (0.03-0.14)	-1.96 (-2.27--1.64)
Burundi	1.84	19.47	9.58	0.03	0.15	4.68

	(0.6-3.73)	(7.06-36.32)		(0.01-0.07)	(0.05-0.27)	(3.92-5.44)
Cambodia	1639.58 (668.91-2711.52)	3595.57 (1669.22-5905.07)	1.19	15.96 (6.51-26.4)	21.1 (9.79-34.65)	-0.82 (-0.92--0.73)
Cameroon	15.95 (6.56-27.42)	21.75 (7.65-44.61)	0.36	0.15 (0.06-0.26)	0.07 (0.02-0.14)	-3.73 (-4.16--3.29)
Canada	1946.06 (876.02-2964.63)	1393.85 (655.19-2240.43)	-0.28	7.14 (3.21-10.88)	3.72 (1.75-5.98)	-3.24 (-3.52--2.96)
Cape Verde	1.3 (0.62-2.04)	4.21 (1.74-6.7)	2.24	0.37 (0.18-0.58)	0.75 (0.31-1.2)	0.63 (0.02-1.25)
Central African Republic	17.82 (7.43-31.11)	35.62 (14.37-67.71)	1	0.65 (0.27-1.14)	0.65 (0.26-1.23)	-0.07 (-0.44-0.3)
Chad	19.7 (8.83-32.99)	26.61 (10.72-47.96)	0.35	0.33 (0.15-0.55)	0.15 (0.06-0.27)	-1.42 (-1.67--1.16)
Chile	619.97 (276.54-953.18)	884.17 (404.95-1421.2)	0.43	4.67 (2.08-7.17)	4.7 (2.15-7.56)	-1.56 (-1.78--1.34)
China	68081.5 (29789.69-111751.1)	48099.59 (18866.72-84760.77)	-0.29	5.79 (2.53-9.5)	3.38 (1.33-5.96)	-3.77 (-3.86--3.67)
Colombia	829.94 (368.4-1234.85)	1776.07 (787.67-2831.03)	1.14	2.55 (1.13-3.8)	3.62 (1.61-5.77)	-0.3 (-0.8-0.2)
Comoros	2.58 (0.88-4.15)	8.72 (3.49-14.68)	2.38	0.56 (0.19-0.9)	1.17 (0.47-1.97)	1.52 (1.3-1.74)
Congo	43.46 (17.58-70.78)	135.71 (57.91-221.38)	2.12	1.81 (0.73-2.95)	2.52 (1.07-4.11)	0.13 (-0.06-0.32)
Cook Islands	0.02 (0-0.03)	0.01 (0-0.01)	-0.5	0.08 (0.03-0.17)	0.03 (0.01-0.08)	-4.86 (-5.11--4.61)
Costa Rica	102.52 (45.97-152.46)	444.34 (186.42-695.29)	3.33	3.37 (1.51-5.01)	9.36 (3.93-14.64)	1.46 (1.26-1.66)
Cote d'Ivoire	3.41 (1.2-6.14)	4.33 (1.35-9.49)	0.27	0.03 (0.01-0.05)	0.02 (0-0.03)	-3.22 (-3.47--2.96)

Croatia	581.68 (257.83-882.4)	884.3 (410.26-1415.76)	0.52	11.97 (5.3-18.15)	21.01 (9.75-33.64)	-0.09 (-0.29-0.12)
Cuba	698.02 (317.69-1080.2)	119.78 (45.44-220.27)	-0.83	6.44 (2.93-9.96)	1.06 (0.4-1.95)	-8.94 (-10.34--7.52)
Cyprus	56.57 (25.34-89.94)	131.03 (57.41-207.03)	1.32	7.27 (3.26-11.56)	9.65 (4.23-15.25)	-0.16 (-0.38-0.05)
Czech Republic	1445.6 (665.71-2202.92)	1317.78 (560.71-2135.96)	-0.09	14.04 (6.47-21.4)	12.39 (5.27-20.09)	-1.91 (-2.23--1.58)
Democratic People's Republic of Korea	481.88 (204.54-849.93)	1418.36 (605.61-2566.17)	1.94	2.34 (0.99-4.13)	5.37 (2.29-9.72)	1.64 (1.42-1.87)
Democratic Republic of the Congo	81.77 (33.17-143.2)	696.11 (273.22-1319.37)	7.51	0.21 (0.09-0.38)	0.77 (0.3-1.47)	4.25 (3.37-5.14)
Denmark	447.22 (203.16-689.67)	406.84 (181.65-673.76)	-0.09	8.69 (3.95-13.41)	6.95 (3.1-11.51)	-2 (-2.22--1.78)
Djibouti	23.06 (9.54-37.85)	44.72 (18.45-82.9)	0.94	5.57 (2.3-9.14)	3.55 (1.47-6.59)	-3.51 (-3.84--3.19)
Dominica	0.6 (0.26-0.98)	0.54 (0.23-0.94)	-0.1	0.83 (0.36-1.36)	0.81 (0.34-1.41)	-1.47 (-2--0.94)
Dominican Republic	167.24 (74.03-263.99)	312.13 (141.5-493.5)	0.87	2.34 (1.04-3.69)	2.83 (1.28-4.48)	-1.43 (-1.75--1.1)
Ecuador	227.38 (100.32-348.05)	1332.29 (560.04-2136.91)	4.86	2.28 (1.01-3.49)	7.37 (3.1-11.83)	2.54 (1.94-3.15)
Egypt	80.68 (33.27-139.99)	120.24 (42.85-227.19)	0.49	0.15 (0.06-0.25)	0.11 (0.04-0.22)	-0.68 (-1.24--0.11)
El Salvador	12.71 (5.43-20.79)	34.86 (13.87-62.18)	1.74	0.24 (0.1-0.39)	0.54 (0.22-0.96)	1.51 (1.33-1.68)
Equatorial Guinea	6.13 (2.44-10.79)	2.91 (1.05-5.75)	-0.53	1.45 (0.58-2.55)	0.19 (0.07-0.38)	-7.37 (-8.5--6.22)
Eritrea	21.14	36.81	0.74	0.62	0.56	-0.73

	(8.08-38.02)	(14.52-68.09)		(0.24-1.12)	(0.22-1.03)	(-0.95--0.51)
Estonia	52.93 (23.61-84.61)	39.47 (17.45-69.94)	-0.25	3.37 (1.51-5.39)	3.01 (1.33-5.34)	-2.98 (-3.35--2.6)
Ethiopia	503.01 (202.23-846.13)	272.14 (112.29-481.77)	-0.46	0.99 (0.4-1.67)	0.25 (0.1-0.44)	-5.57 (-5.93--5.2)
Fiji	7.25 (3.15-11.76)	5.19 (1.88-9.48)	-0.28	0.96 (0.42-1.55)	0.56 (0.2-1.03)	-2.72 (-3.46--1.99)
Finland	348.49 (159.93-542.66)	299.58 (127.36-500.77)	-0.14	6.96 (3.19-10.83)	5.41 (2.3-9.05)	-3.02 (-3.26--2.78)
France	6353.42 (2871.18-9913.77)	6959.53 (3009.9-11233.1)	0.1	11 (4.97-17.16)	10.48 (4.53-16.92)	-1.14 (-1.22--1.05)
Gabon	10.34 (3.8-19)	14.52 (6.04-24.94)	0.4	1.05 (0.39-1.93)	0.8 (0.33-1.37)	-1.08 (-1.18--0.98)
Georgia	90.72 (39.74-146.15)	139.16 (59.13-229.13)	0.53	1.64 (0.72-2.65)	3.86 (1.64-6.35)	1.84 (1.3-2.38)
Germany	6955.1 (3209.91-11207.89)	6361.17 (2786.98-10268.71)	-0.09	8.7 (4.02-14.02)	7.45 (3.26-12.03)	-2.14 (-2.4--1.89)
Ghana	11.42 (4.37-20.2)	1 (0.2-2.39)	-0.91	0.08 (0.03-0.13)	0 (0-0.01)	-11.89 (-13.31--10.45)
Greece	196.19 (85.82-316.41)	467.11 (202.64-792.95)	1.38	1.89 (0.83-3.05)	4.59 (1.99-7.79)	1.24 (0.97-1.51)
Greenland	4.78 (1.97-7.76)	2.86 (1.11-4.98)	-0.4	8.6 (3.54-13.96)	5.1 (1.99-8.87)	-3.87 (-4.02--3.72)
Grenada	2.88 (1.23-4.52)	4.21 (1.88-6.9)	0.46	3.31 (1.41-5.19)	4.1 (1.83-6.73)	-0.11 (-0.26-0.04)
Guam	0.04 (0.01-0.09)	0.04 (0.01-0.11)	0	0.03 (0.01-0.06)	0.02 (0-0.07)	-2.5 (-2.81--2.19)
Guatemala	8.89 (3.63-15.11)	55.4 (22.35-94.59)	5.23	0.11 (0.04-0.18)	0.35 (0.14-0.6)	1.66 (1.07-2.26)

Guinea	19.19 (8.32-31.04)	35 (13.8-61.54)	0.82	0.32 (0.14-0.52)	0.26 (0.1-0.46)	0.27 (0.01-0.53)
Guinea-Bissau	36.38 (15-57.55)	19.13 (7.5-33.34)	-0.47	3.61 (1.49-5.71)	0.93 (0.36-1.62)	-4.87 (-5.32--4.41)
Guyana	37.78 (16.75-59.63)	28.69 (11.81-46.44)	-0.24	4.85 (2.15-7.65)	3.75 (1.54-6.07)	-1.87 (-2.33--1.41)
Haiti	87.69 (35.21-148.57)	298.36 (126.44-520.24)	2.4	1.37 (0.55-2.33)	2.32 (0.98-4.04)	0.55 (0.13-0.98)
Honduras	9.4 (4.03-15.82)	45.12 (18.12-79.55)	3.8	0.2 (0.09-0.34)	0.45 (0.18-0.79)	1.65 (1.25-2.05)
Hungary	995.53 (432.62-1572.64)	1500.96 (677.88-2381.86)	0.51	9.58 (4.16-15.13)	15.64 (7.06-24.82)	0.02 (-0.47-0.52)
Iceland	29.79 (12.99-46.47)	33.4 (14.58-54.2)	0.12	11.73 (5.12-18.3)	9.53 (4.16-15.47)	-2 (-2.21--1.79)
India	11793.12 (5438.96-18256.37)	17209.84 (7656.84-26287.45)	0.46	1.38 (0.64-2.14)	1.22 (0.54-1.86)	-1.9 (-2.18--1.63)
Indonesia	11851.93 (5202.91-18634.03)	20050.43 (8609.09-33214.18)	0.69	6.41 (2.81-10.07)	7.19 (3.09-11.91)	-1.02 (-1.17--0.87)
Iran	358.85 (160.82-567.9)	515.03 (214.73-825.43)	0.44	0.63 (0.28-0.99)	0.6 (0.25-0.97)	-1.74 (-2.19--1.29)
Iraq	41.32 (17.58-69.02)	367.09 (156.46-620.36)	7.88	0.22 (0.1-0.37)	0.89 (0.38-1.5)	2.98 (2.17-3.81)
Ireland	146.94 (64.99-235.5)	154.5 (64.88-261.74)	0.05	4.08 (1.8-6.54)	3.13 (1.31-5.3)	-1.41 (-1.66--1.16)
Israel	66.8 (29.25-112.62)	100.07 (42.57-173.67)	0.5	1.35 (0.59-2.27)	1.04 (0.44-1.81)	-1.95 (-2.12--1.78)
Italy	3589.31 (1628.82-5556.81)	4677.88 (2039.56-7191.17)	0.3	6.32 (2.87-9.78)	7.82 (3.41-12.02)	-0.53 (-0.77--0.29)
Jamaica	24.06 (10.61-38.6	64.78 (25-111.45)	1.69	1.02 (0.45-1.63)	2.31 (0.89-3.98)	1.84 (1.53-2.

	6)					16)
Japan	6446.1 (2768.13-10156.63)	13423.52 (6034.1-20613.39)	1.08	5.12 (2.2-8.07)	10.51 (4.73-16.14)	0.7 (0.52-0.88)
Jordan	55.27 (24.95-87.38)	164.42 (65.98-276.03)	1.97	1.48 (0.67-2.34)	1.33 (0.54-2.24)	-2.57 (-2.81--2.33)
Kazakhstan	659.51 (270.13-1025.3)	376.24 (173.94-608.96)	-0.43	4.02 (1.65-6.25)	1.98 (0.92-3.21)	-3.75 (-4.58--2.92)
Kenya	49.72 (21.08-81.92)	122.89 (56.06-203.45)	1.47	0.21 (0.09-0.35)	0.25 (0.11-0.41)	-0.15 (-0.28--0.02)
Kiribati	0.46 (0.19-0.76)	0.71 (0.29-1.26)	0.54	0.62 (0.26-1.02)	0.59 (0.24-1.04)	-1.21 (-1.36--1.06)
Kuwait	10.75 (4.74-16.9)	46.23 (20.37-73.81)	3.3	0.63 (0.28-0.98)	0.99 (0.44-1.59)	-0.13 (-0.52-0.25)
Kyrgyzstan	107.42 (46.83-168.02)	64.25 (28.23-103.95)	-0.4	2.41 (1.05-3.76)	0.94 (0.41-1.51)	-4.26 (-4.69--3.83)
Lao People's Democratic Republic	696 (257.08-1156.23)	850.58 (338.61-1435.14)	0.22	16.69 (6.17-27.73)	11.53 (4.59-19.45)	-2.37 (-2.5--2.23)
Latvia	137.05 (60.91-218.92)	131.85 (54.88-210.11)	-0.04	5.16 (2.29-8.24)	7.05 (2.93-11.23)	-1.15 (-1.68--0.63)
Lebanon	10.8 (4.21-19.62)	38.56 (15.39-68.47)	2.57	0.36 (0.14-0.66)	0.7 (0.28-1.24)	2.03 (1.46-2.61)
Lesotho	1.13 (0.39-2.17)	1.58 (0.49-3.41)	0.4	0.07 (0.03-0.14)	0.08 (0.03-0.18)	0.54 (0.37-0.72)
Liberia	21.87 (10.24-35.29)	82.71 (34.13-155.19)	2.78	0.89 (0.42-1.43)	1.51 (0.63-2.84)	1.7 (1.34-2.05)
Libya	26.06 (11.61-42.66)	131.33 (55.77-224.86)	4.04	0.62 (0.28-1.01)	1.91 (0.81-3.27)	1.7 (1.36-2.05)
Lithuania	41.18 (17.18-70.08)	77.65 (33.56-132.97)	0.89	1.12 (0.47-1.91)	2.85 (1.23-4.87)	1.43 (0.88-1.98)
Luxembourg	43.53	36.02	-0.17	11.42	5.59	-2.89

	(19.83-66.94)	(15.26-58.15)		(5.2-17.56)	(2.37-9.03)	(-3.14--2.63)
Macedonia	44.98 (20.2-70.12)	70.93 (29.16-120.37)	0.58	2.26 (1.01-3.52)	3.26 (1.34-5.53)	-0.67 (-1.12--0.22)
Madagascar	132.59 (58.71-213.41)	314.83 (127.97-524.72)	1.37	1.11 (0.49-1.79)	1.1 (0.45-1.84)	-0.22 (-0.36--0.08)
Malawi	3.75 (1.4-6.91)	4.91 (1.58-10.32)	0.31	0.04 (0.01-0.07)	0.03 (0.01-0.05)	-2.82 (-3.41--2.24)
Malaysia	1933.34 (895.43-3061.67)	3218.69 (1434.2-5103.19)	0.66	10.94 (5.07-17.33)	10.12 (4.51-16.04)	-1.83 (-2.16--1.5)
Maldives	4.46 (1.81-7.37)	9.88 (4.11-15.84)	1.22	2.01 (0.82-3.32)	1.91 (0.79-3.06)	-2.66 (-3.05--2.27)
Mali	36.36 (16.45-57.32)	25.7 (9.58-45.36)	-0.29	0.42 (0.19-0.66)	0.11 (0.04-0.19)	-3.89 (-4.47--3.3)
Malta	18.77 (8.53-29.61)	18.08 (7.66-28.91)	-0.04	5.06 (2.3-7.99)	4.09 (1.73-6.54)	-1.97 (-2.55--1.39)
Marshall Islands	0.14 (0.06-0.26)	0.23 (0.09-0.45)	0.64	0.31 (0.13-0.57)	0.41 (0.16-0.8)	-0.89 (-0.98--0.8)
Mauritania	14.83 (6.09-23.46)	13.38 (5.42-23.72)	-0.1	0.72 (0.3-1.14)	0.3 (0.12-0.54)	-2.64 (-3.08--2.21)
Mauritius	75.77 (34.61-112.72)	93.66 (39.4-147.11)	0.24	6.91 (3.16-10.29)	7.36 (3.1-11.57)	-2.77 (-3.35--2.19)
Mexico	100.84 (42.76-171.57)	593.85 (254.7-1050.03)	4.89	0.12 (0.05-0.2)	0.46 (0.2-0.81)	2.83 (2.45-3.2)
Micronesia (Federated States of)	0.47 (0.18-0.89)	0.5 (0.19-0.99)	0.06	0.45 (0.17-0.86)	0.49 (0.19-0.97)	-1 (-1.1--0.9)
Moldova	64.36 (26.79-107.96)	132.33 (57.46-231.23)	1.06	1.45 (0.6-2.43)	3.68 (1.6-6.43)	1.58 (1.13-2.03)
Monaco	0.99 (0.4-1.86)	1.02 (0.37-1.88)	0.03	3.26 (1.3-6.11)	2.69 (0.98-4.98)	-0.67 (-0.91--0.42)

Mongolia	168.41 (69.68-268.78)	229.33 (100.25-360.25)	0.36	7.8 (3.23-12.46)	6.87 (3-10.8)	-2.32 (-2.55--2.08)
Montenegro	9.26 (4.25-15.01)	14.78 (5.84-24.71)	0.6	1.48 (0.68-2.4)	2.39 (0.94-4)	-0.87 (-1.46--0.27)
Morocco	13.73 (5.13-27.11)	32.37 (10.57-65.63)	1.36	0.05 (0.02-0.11)	0.09 (0.03-0.18)	-0.04 (-0.15-0.08)
Mozambique	45.64 (19.51-71.79)	36.66 (14.59-62.76)	-0.2	0.34 (0.15-0.54)	0.12 (0.05-0.2)	-2.79 (-3.24--2.33)
Myanmar	6817.44 (2790.19-11757.8)	6122.65 (2657.77-9739.7)	-0.1	16.86 (6.9-29.08)	10.85 (4.71-17.26)	-3.31 (-3.59--3.02)
Namibia	16.37 (7.4-25.91)	33.48 (15.12-57.49)	1.05	1.17 (0.53-1.85)	1.38 (0.62-2.36)	-0.22 (-0.36--0.08)
Nauru	0.01 (0-0.03)	0.02 (0.01-0.05)	1	0.14 (0.04-0.31)	0.21 (0.07-0.42)	0.6 (-0.72-1.94)
Nepal	280.37 (117.57-487.55)	185.05 (77.49-316.23)	-0.34	1.44 (0.6-2.5)	0.59 (0.25-1.02)	-4.21 (-4.85--3.55)
Netherlands	2072.07 (935.01-3147.3)	1776.12 (806.76-2921.31)	-0.14	13.89 (6.27-21.09)	10.32 (4.69-16.97)	-2.66 (-2.97--2.36)
New Zealand	299.59 (136.14-482.76)	380.74 (161.29-618.55)	0.27	8.77 (3.98-14.13)	7.37 (3.12-11.97)	-0.98 (-1.26--0.69)
Nicaragua	24.38 (10.69-38.07)	53.61 (23.32-87.4)	1.2	0.63 (0.27-0.98)	0.8 (0.35-1.31)	-1.83 (-2.15--1.52)
Niger	20.83 (9.36-34.33)	23.88 (9.3-45.73)	0.15	0.26 (0.12-0.43)	0.1 (0.04-0.18)	-3.6 (-4.06--3.13)
Nigeria	192.62 (84.14-330.06)	132.64 (52.24-231.76)	-0.31	0.21 (0.09-0.37)	0.06 (0.02-0.1)	-3.84 (-4.28--3.4)
Niue	0.01 (0-0.01)	0 (0-0.01)	-1	0.3 (0.11-0.6)	0.22 (0.07-0.44)	-2.26 (-2.44--2.08)
Northern Mariana	0.01 (0-0.03)	0.04 (0.01-0.1)	3	0.03 (0.01-0.08)	0.08 (0.02-0.21)	1.74 (1.21-2.

Islands						28)
Norway	664.26 (302.93-1012.66)	456.99 (205.29-724.34)	-0.31	15.64 (7.13-23.85)	8.43 (3.79-13.37)	-3.28 (-3.51--3.05)
Oman	7.13 (3.11-11.84)	6.69 (2.72-11.53)	-0.06	0.36 (0.16-0.6)	0.14 (0.06-0.25)	-3.37 (-3.92--2.81)
Pakistan	2246.34 (1017.97-3535.35)	5322.62 (2345.47-8735.48)	1.37	2.02 (0.92-3.18)	2.26 (1-3.71)	0 (-0.28-0.28)
Palau	0.03 (0.01-0.06)	0.03 (0.01-0.08)	0	0.2 (0.06-0.43)	0.18 (0.05-0.42)	-1.61 (-1.78--1.43)
Palestine	95.71 (43.77-170.65)	242.01 (102.78-392.94)	1.53	4.68 (2.14-8.34)	4.71 (2-7.65)	-0.25 (-0.54-0.04)
Panama	133.37 (59.35-200.94)	397.81 (171.43-634.39)	1.98	5.58 (2.48-8.41)	9.27 (3.99-14.78)	0.24 (-0.02-0.5)
Papua New Guinea	2.69 (0.94-5.17)	5.69 (2.15-11.41)	1.12	0.07 (0.02-0.13)	0.05 (0.02-0.11)	-1.48 (-1.66--1.3)
Paraguay	11.69 (4.84-19.26)	42.04 (16.97-74.45)	2.6	0.29 (0.12-0.48)	0.59 (0.24-1.04)	1.26 (1.03-1.49)
Peru	519.22 (221.73-813.27)	358.74 (157.42-608.15)	-0.31	2.4 (1.02-3.76)	0.99 (0.43-1.68)	-5.19 (-5.82--4.55)
Philippines	4908.04 (2240.59-7541.45)	15192 (6936.14-23871.18)	2.1	7.79 (3.56-11.97)	13.41 (6.12-21.08)	0.65 (0.45-0.84)
Poland	920.44 (410.47-1461.07)	1549.12 (604.11-2561.66)	0.68	2.41 (1.08-3.83)	4.05 (1.58-6.7)	0.09 (-0.29-0.47)
Portugal	420.43 (193.68-673.13)	703.82 (315.2-1132.24)	0.67	4.15 (1.91-6.64)	6.63 (2.97-10.67)	0.35 (0.01-0.69)
Puerto Rico	163.82 (71.72-269.01)	200.69 (85.35-347.97)	0.23	4.53 (1.99-7.45)	6.09 (2.59-10.56)	-1.24 (-1.35--1.13)
Qatar	0.52 (0.18-1.01)	1.73 (0.52-3.35)	2.33	0.12 (0.04-0.23)	0.06 (0.02-0.11)	-3.83 (-4.27--3.4)
Romania	582.62	574.87	-0.01	2.49	3.04	-1.7

	(253.85-918.01)	(232.15-971.7)		(1.09-3.93)	(1.23-5.13)	(-2.05--1.34)
Russian Federation	7125.77 (3304.01-10930.51)	9841.4 (4520.31-15307.37)	0.38	4.72 (2.19-7.24)	6.79 (3.12-10.57)	-1.06 (-1.7--0.41)
Rwanda	0.95 (0.27-2.16)	1.94 (0.53-4.66)	1.04	0.01 (0-0.03)	0.01 (0-0.04)	-3.59 (-4.77--2.39)
Saint Kitts and Nevis	8.55 (3.81-12.99)	14.57 (6.26-23.64)	0.7	20.63 (9.19-31.33)	24.85 (10.68-40.31)	0.23 (0.07-0.39)
Saint Lucia	3.12 (1.4-4.81)	13.37 (5.62-21.56)	3.29	2.29 (1.02-3.52)	7.53 (3.17-12.15)	1.7 (1.39-2.01)
Saint Vincent and the Grenadines	3.13 (1.38-4.85)	5.17 (2.31-8.41)	0.65	2.86 (1.26-4.43)	4.53 (2.02-7.37)	-1.27 (-1.6--0.95)
Samoa	0.06 (0.02-0.13)	0.09 (0.03-0.2)	0.5	0.04 (0.01-0.08)	0.04 (0.01-0.09)	-2.71 (-3.47--1.94)
San Marino	1.55 (0.65-2.63)	1.56 (0.61-2.74)	0.01	6.52 (2.73-11.08)	4.77 (1.87-8.36)	-1.55 (-2.01--1.08)
Sao Tome and Principe	0.11 (0.04-0.2)	0.12 (0.04-0.25)	0.09	0.09 (0.03-0.16)	0.06 (0.02-0.12)	-0.81 (-1.6--0.02)
Saudi Arabia	40.77 (17.93-70.24)	169.95 (65.95-300.84)	3.17	0.26 (0.11-0.44)	0.45 (0.17-0.8)	0.91 (0.54-1.29)
Senegal	62.78 (27.43-99.51)	19.48 (7.27-36.83)	-0.69	0.82 (0.36-1.3)	0.12 (0.05-0.23)	-7.29 (-8.28--6.28)
Serbia	534.15 (238.11-841.51)	647.34 (256.44-1071.77)	0.21	5.55 (2.47-8.74)	7.26 (2.88-12.02)	-1.69 (-2.13--1.25)
Seychelles	5.77 (2.6-8.98)	6.11 (2.92-9.91)	0.06	7.92 (3.57-12.32)	5.8 (2.77-9.4)	-1.9 (-2.05--1.75)
Sierra Leone	147.26 (68.61-231.12)	165.59 (66.73-273.66)	0.12	3.55 (1.65-5.57)	1.87 (0.75-3.09)	-1.79 (-1.91--1.67)
Singapore	270.71 (124.86-433.91)	353.03 (150.93-574.61)	0.3	8.88 (4.1-14.24)	6.16 (2.64-10.03)	-3.3 (-3.49--3.11)

Slovakia	554.97 (254.01-861.01)	1081.93 (446.2-1781.1)	0.95	10.5 (4.81-16.3)	19.93 (8.22-32.8)	0.79 (0.18-1.4)
Slovenia	116.85 (49.92-182.63)	116.61 (50.68-193.25)	0	5.92 (2.53-9.25)	5.63 (2.45-9.34)	-2.24 (-2.43--2.04)
Solomon Islands	0.26 (0.07-0.55)	0.77 (0.25-1.59)	1.96	0.08 (0.02-0.16)	0.11 (0.04-0.23)	-0.06 (-0.26-0.15)
Somalia	239.87 (98.64-438.59)	787.04 (334.16-1409.17)	2.28	3.02 (1.24-5.52)	3.64 (1.55-6.52)	0.52 (0.12-0.92)
South Africa	189.84 (84.6-296.81)	643.4 (281.59-1017.85)	2.39	0.51 (0.23-0.8)	1.13 (0.5-1.79)	1.84 (1.55-2.14)
South Korea	2203.01 (963.95-3440.35)	8823.22 (4060.98-14507.14)	3.01	4.98 (2.18-7.78)	17.11 (7.87-28.13)	0.97 (0.55-1.39)
South Sudan	8.71 (3.17-18.27)	14.49 (5.24-28.08)	0.66	0.15 (0.05-0.31)	0.15 (0.05-0.29)	-0.2 (-0.37--0.03)
Spain	1892.83 (849.03-2901.23)	4262.49 (1823.14-6743.85)	1.25	4.88 (2.19-7.48)	9.36 (4-14.81)	1.23 (0.92-1.55)
Sri Lanka	148.21 (67.93-226.72)	179.17 (68.46-314.35)	0.21	0.87 (0.4-1.32)	0.8 (0.31-1.41)	-2.11 (-2.47--1.75)
Sudan	318.72 (119.68-568.47)	520.46 (197.53-961.92)	0.63	1.59 (0.6-2.84)	1.2 (0.45-2.22)	-1.22 (-1.27--1.16)
Suriname	41.45 (18.63-62.84)	101.19 (44-160.59)	1.44	10.72 (4.82-16.25)	17.47 (7.6-27.72)	0.11 (-0.17-0.39)
Swaziland	4.33 (1.78-7.47)	16.41 (6.31-29.19)	2.79	0.54 (0.22-0.93)	1.42 (0.55-2.53)	2.55 (1.88-3.23)
Sweden	1146.43 (507.1-1764.05)	721.4 (301.03-1178.41)	-0.37	13.35 (5.9-20.54)	6.95 (2.9-11.36)	-3.17 (-3.35--2.98)
Switzerland	709.06 (308.51-1085.15)	887.42 (397.46-1422.62)	0.25	10.33 (4.49-15.8)	9.95 (4.45-15.94)	-1.15 (-1.53--0.76)
Syria	75.53 (31.56-121.	164.35 (68.2-288.8	1.18	0.59 (0.25-0.96)	1.17 (0.49-2.06)	-0.94 (-1.4--0.

	79)	5)				47)
Taiwan (Province of China)	1811.91 (814.83-2732.21)	3097.71 (1313.78-4868.94)	0.71	8.89 (4-13.4)	13.11 (5.56-20.6)	-0.62 (-0.84--0.4)
Tajikistan	176.27 (78.05-269.94)	193.13 (80.2-316.78)	0.1	3.28 (1.45-5.03)	1.9 (0.79-3.12)	-3.39 (-3.78--2.99)
Tanzania	60 (24.92-102.65)	116.9 (46.62-206.56)	0.95	0.23 (0.1-0.4)	0.2 (0.08-0.35)	-1.12 (-1.33--0.9)
Thailand	8546.75 (3823.62-13195.56)	17234.58 (7117.51-28834.77)	1.02	15.06 (6.74-23.25)	25.85 (10.67-43.24)	-1.2 (-1.4--1)
The Bahamas	16.87 (7.34-25.39)	51.38 (22.94-81.8)	2.05	6.57 (2.86-9.89)	13.24 (5.91-21.08)	0.66 (0.44-0.89)
The Gambia	7.75 (3.48-11.99)	4.49 (1.88-7.54)	-0.42	0.79 (0.35-1.22)	0.19 (0.08-0.31)	-6.01 (-6.88--5.13)
Timor-Leste	10.72 (4.57-17.75)	26.23 (11.18-42.42)	1.45	1.37 (0.58-2.27)	1.88 (0.8-3.04)	-0.44 (-0.78--0.09)
Togo	2.28 (0.85-4.06)	4.78 (1.53-9.33)	1.1	0.06 (0.02-0.11)	0.06 (0.02-0.11)	-1.22 (-1.72--0.71)
Tokelau	0.01 (0-0.01)	0 (0-0.01)	-1	0.5 (0.2-0.91)	0.26 (0.09-0.53)	-3.12 (-3.23--3.01)
Tonga	0.21 (0.08-0.37)	0.19 (0.07-0.36)	-0.1	0.21 (0.08-0.37)	0.18 (0.07-0.34)	-1.36 (-1.45--1.28)
Trinidad and Tobago	58.23 (25.61-88.6)	75.02 (32.16-125.67)	0.29	4.83 (2.13-7.35)	5.38 (2.31-9.02)	-2.9 (-3.31--2.49)
Tunisia	26.88 (10.8-44.94)	45.4 (17.87-86.15)	0.69	0.32 (0.13-0.54)	0.38 (0.15-0.73)	-2.11 (-2.38--1.84)
Turkey	180.7 (70.58-338.89)	318 (121.11-609.3)	0.76	0.31 (0.12-0.59)	0.38 (0.14-0.73)	-0.68 (-0.86--0.49)
Turkmenistan	98.27 (43.86-149.48)	75.62 (34.29-129.19)	-0.23	2.66 (1.19-4.04)	1.47 (0.66-2.5)	-4.07 (-4.53--3.6)
Tuvalu	0.07	0.05	-0.29	0.69	0.43	-1.74

	(0.03-0.12)	(0.02-0.1)		(0.28-1.31)	(0.15-0.83)	(-1.94--1.55)
Uganda	20.61 (7.63-37.11)	60.6 (20.67-109.47)	1.94	0.12 (0.04-0.21)	0.14 (0.05-0.25)	0.14 (-0.07-0.35)
Ukraine	1784.89 (794.3-2810.39)	1883.04 (804.58-3286.17)	0.05	3.39 (1.51-5.33)	4.37 (1.87-7.63)	-1.24 (-1.86--0.62)
United Arab Emirates	8.82 (3.2-15.76)	44.57 (17.68-79)	4.05	0.47 (0.17-0.84)	0.46 (0.18-0.82)	1.24 (0.63-1.86)
United Kingdom	9084.22 (3994.41-14330.47)	6365.78 (2893.04-10196.18)	-0.3	15.85 (6.97-25.01)	9.38 (4.26-15.03)	-2.2 (-2.53--1.86)
United States	28250.24 (12888.31-42745.31)	18261.25 (8373.21-28368.23)	-0.35	11.12 (5.07-16.82)	5.49 (2.52-8.53)	-3.12 (-3.32--2.92)
Uruguay	608.65 (272.59-913.87)	344.01 (155.71-550.8)	-0.43	19.39 (8.68-29.11)	10.1 (4.57-16.17)	-3.31 (-3.54--3.07)
Uzbekistan	431.2 (187.92-658.03)	294.43 (124.32-473.96)	-0.32	2.06 (0.9-3.14)	0.86 (0.36-1.38)	-5.37 (-6.02--4.72)
Vanuatu	0.34 (0.12-0.65)	0.52 (0.19-1)	0.53	0.22 (0.08-0.43)	0.16 (0.06-0.32)	-2.64 (-2.95--2.34)
Venezuela	363.12 (157.51-541.74)	1422.56 (608.09-2394.59)	2.92	1.93 (0.84-2.88)	5.34 (2.28-8.99)	0.68 (0.39-0.98)
Viet Nam	7692.93 (3342.56-11895.11)	18148.8 (8161.69-29470)	1.36	11.28 (4.9-17.43)	18.1 (8.14-29.39)	-0.29 (-0.44--0.14)
Virgin Islands, U.S.	5.88 (2.31-9.48)	4.05 (1.6-7.08)	-0.31	5.54 (2.17-8.94)	4.72 (1.86-8.24)	-2.83 (-3.22--2.43)
Yemen	140.28 (61.26-242.43)	538.94 (232.07-1011.29)	2.84	1.03 (0.45-1.78)	1.6 (0.69-3.01)	0.01 (-0.32-0.34)
Zambia	16.05 (6.56-29.11)	66.49 (22.21-158.2)	3.14	0.2 (0.08-0.37)	0.34 (0.11-0.81)	1.14 (0.61-1.67)
Zimbabwe	41.04 (18.33-67.14)	90.2 (35.34-153.68)	1.2	0.4 (0.18-0.65)	0.58 (0.23-0.99)	0.28 (-0.23-0.79)

Supplementary table 1. The DALYs of CRC-LFD cases and rates in 1990 and 2021 across 204 countries, and the trends from 1990 to 2021.

Location	1990_Death cases (95% UI)	2021_Death cases (95% UI)	Percentage change	1990_ASMR_per 100000(95% UI)	2021_ASMR_per 100 000(95% UI)	EAPC (95% CI)
Afghanistan	8.53 (2.65-16.46)	31.07 (11.52-59.29)	2.64	0.09 (0.03-0.17)	0.1 (0.04-0.19)	1.79 (0.94-2.65)
Albania	1.68 (0.73-2.63)	1.91 (0.71-3.4)	0.14	0.05 (0.02-0.08)	0.07 (0.03-0.13)	-3.35 (-3.79--2.91)
Algeria	5.8 (2.66-9.01)	5.3 (2.1-9.09)	-0.09	0.02 (0.01-0.04)	0.01 (0-0.02)	-4.29 (-4.75--3.83)
American Samoa	0 (0-0.01)	0 (0-0.01)	NA	0.01 (0-0.01)	0.01 (0-0.01)	-0.96 (-1.22--0.7)
Andorra	0.1 (0.03-0.19)	0.2 (0.07-0.38)	1	0.19 (0.06-0.34)	0.24 (0.09-0.44)	-1.12 (-1.51--0.73)
Angola	4.12 (1.77-6.8)	3.06 (1.32-5.31)	-0.26	0.04 (0.02-0.07)	0.01 (0-0.02)	-5.51 (-6.08--4.93)
Antigua and Barbuda	0.46 (0.2-0.7)	0.67 (0.31-1.03)	0.46	0.76 (0.33-1.16)	0.75 (0.34-1.15)	-0.65 (-0.81--0.48)
Argentina	142 (64.62-215.68)	220.51 (99.32-350.82)	0.55	0.43 (0.2-0.65)	0.48 (0.22-0.77)	0.23 (-0.08-0.53)
Armenia	5.14 (2.31-7.92)	4.68 (2-7.48)	-0.09	0.15 (0.07-0.23)	0.16 (0.07-0.25)	-3.04 (-3.53--2.54)
Australia	88.42 (39.68-138.91)	97.43 (43.14-163.06)	0.1	0.52 (0.24-0.82)	0.38 (0.17-0.63)	-3.25 (-3.56--2.93)
Austria	33.49 (15.57-52.47)	16.87 (7.16-29.49)	-0.5	0.43 (0.2-0.68)	0.19 (0.08-0.33)	-3.88 (-4.12--3.64)
Azerbaijan	6.24 (2.88-9.89)	3.44 (1.35-5.64)	-0.45	0.09 (0.04-0.14)	0.03 (0.01-0.05)	-5.62 (-6.25--4.99)
Bahrain	0.05 (0.02-0.09)	0.14 (0.05-0.26)	1.8	0.01 (0-0.02)	0.01 (0-0.02)	-1.72 (-1.97--1.47)
Bangladesh	204.26	371.92	0.82	0.19	0.23 (0.1-0.37)	-1.62

	(93.42-327.71)	(167.7-611.81)		(0.09-0.3)		(-1.79--1.46)
Barbados	1.36 (0.57-2.14)	2.23 (0.91-3.77)	0.64	0.54 (0.23-0.84)	0.74 (0.3-1.26)	0.37 (0.2-0.54)
Belarus	3.12 (1.25-5.68)	6.26 (2.58-11.95)	1.01	0.03 (0.01-0.05)	0.07 (0.03-0.13)	0.44 (-0.42-1.31)
Belgium	58.95 (26.58-90.46)	46.23 (20.06-77.72)	-0.22	0.59 (0.27-0.91)	0.4 (0.17-0.68)	-2.24 (-2.37--2.11)
Belize	0.06 (0.02-0.09)	0.16 (0.07-0.26)	1.67	0.03 (0.01-0.05)	0.04 (0.02-0.06)	-0.08 (-0.42-0.25)
Benin	0.1 (0.04-0.19)	0.18 (0.07-0.36)	0.8	0 (0-0)	0 (0-0)	-0.48 (-1.06-0.1)
Bermuda	0.16 (0.07-0.25)	0.63 (0.27-1.02)	2.94	0.27 (0.12-0.42)	0.99 (0.42-1.61)	1.66 (1.24-2.08)
Bhutan	0.44 (0.19-0.73)	0.53 (0.23-0.95)	0.2	0.07 (0.03-0.12)	0.07 (0.03-0.13)	-2.49 (-2.61--2.38)
Bolivia	5.13 (2.18-8.41)	15.84 (6.61-27.04)	2.09	0.08 (0.03-0.13)	0.13 (0.06-0.23)	0.13 (-0.13-0.39)
Bosnia and Herzegovina	0.75 (0.3-1.31)	1.29 (0.47-2.44)	0.72	0.02 (0.01-0.03)	0.04 (0.01-0.07)	-0.23 (-0.69-0.24)
Botswana	0.38 (0.16-0.61)	0.99 (0.41-1.7)	1.61	0.03 (0.01-0.05)	0.04 (0.02-0.07)	-0.39 (-0.89-0.11)
Brazil	147.62 (65.82-224.15)	336.3 (151.9-526.06)	1.28	0.1 (0.04-0.15)	0.15 (0.07-0.24)	-1.35 (-1.58--1.12)
Brunei	0.36 (0.15-0.57)	1.55 (0.67-2.51)	3.31	0.14 (0.06-0.22)	0.34 (0.15-0.56)	2.14 (1.77-2.51)
Bulgaria	32.65 (14.67-51.2)	62.83 (26.69-103.38)	0.92	0.38 (0.17-0.59)	0.93 (0.39-1.52)	1.03 (0.29-1.78)
Burkina Faso	0.55 (0.22-0.93)	0.69 (0.26-1.27)	0.25	0.01 (0-0.01)	0 (0-0.01)	-1.88 (-2.16--1.59)

Burundi	0.07 (0.02-0.14)	0.67 (0.24-1.24)	8.57	0 (0-0)	0.01 (0-0.01)	4.42 (3.73-5.12)
Cambodia	54.94 (22.66-89.75)	128.98 (59.56-211)	1.35	0.53 (0.22-0.87)	0.76 (0.35-1.24)	-0.57 (-0.66--0.48)
Cameroon	0.61 (0.25-1.03)	0.82 (0.28-1.67)	0.34	0.01 (0-0.01)	0 (0-0.01)	-3.3 (-3.69--2.92)
Canada	93.68 (41.8-145.92)	77.48 (33.42-134.49)	-0.17	0.34 (0.15-0.54)	0.21 (0.09-0.36)	-3.2 (-3.47--2.93)
Cape Verde	0.07 (0.03-0.11)	0.22 (0.09-0.36)	2.14	0.02 (0.01-0.03)	0.04 (0.02-0.06)	0.96 (0.35-1.58)
Central African Republic	0.57 (0.24-1)	1.1 (0.43-2.05)	0.93	0.02 (0.01-0.04)	0.02 (0.01-0.04)	-0.09 (-0.44-0.27)
Chad	0.81 (0.36-1.36)	1 (0.41-1.74)	0.23	0.01 (0.01-0.02)	0.01 (0-0.01)	-1.3 (-1.55--1.06)
Chile	27.39 (12.41-42.33)	44.24 (20.82-73.8)	0.62	0.21 (0.09-0.32)	0.24 (0.11-0.39)	-1.52 (-1.7--1.33)
China	2100.37 (927.2-3451.54)	1738.8 (693.49-3023.7)	-0.17	0.18 (0.08-0.29)	0.12 (0.05-0.21)	-3.74 (-3.85--3.62)
Colombia	31.87 (14.08-47.85)	74.93 (32.69-121.96)	1.35	0.1 (0.04-0.15)	0.15 (0.07-0.25)	-0.58 (-1.05--0.1)
Comoros	0.09 (0.03-0.14)	0.33 (0.14-0.56)	2.67	0.02 (0.01-0.03)	0.04 (0.02-0.07)	1.76 (1.57-1.95)
Congo	1.49 (0.6-2.42)	4.49 (1.91-7.23)	2.01	0.06 (0.03-0.1)	0.08 (0.04-0.13)	0.18 (0.02-0.35)
Cook Islands	0 (0-0)	0 (0-0)	NA	0 (0-0.01)	0 (0-0)	-4.87 (-5.09--4.66)
Costa Rica	4.31 (1.96-6.46)	18.65 (7.89-29.51)	3.33	0.14 (0.06-0.21)	0.39 (0.17-0.62)	1.24 (1.02-1.46)
Cote d'Ivoire	0.11 (0.04-0.21)	0.15 (0.05-0.31)	0.36	0 (0-0)	0 (0-0)	-2.96 (-3.16--

						2.77)
Croatia	27.14 (11.92-41.55)	46.82 (21.39-77.73)	0.73	0.56 (0.25-0.85)	1.11 (0.51-1.85)	-0.16 (-0.35-0.04)
Cuba	30.9 (13.79-48.36)	7.23 (2.72-13.31)	-0.77	0.28 (0.13-0.45)	0.06 (0.02-0.12)	-8.08 (-9.28--6.87)
Cyprus	2.83 (1.28-4.47)	6.94 (3.04-10.97)	1.45	0.36 (0.17-0.58)	0.51 (0.22-0.81)	-0.33 (-0.54--0.11)
Czech Republic	68.78 (31.32-103.85)	68.05 (29.55-107.43)	-0.01	0.67 (0.3-1.01)	0.64 (0.28-1.01)	-1.87 (-2.2--1.53)
Democratic People's Republic of Korea	16.25 (7-28.02)	51.53 (21.84-92.08)	2.17	0.08 (0.03-0.14)	0.2 (0.08-0.35)	1.43 (1.2-1.67)
Democratic Republic of the Congo	2.81 (1.16-4.93)	23.74 (9.23-44.39)	7.45	0.01 (0-0.01)	0.03 (0.01-0.05)	4.16 (3.3-5.03)
Denmark	23.55 (10.64-36.76)	24.41 (10.55-40.72)	0.04	0.46 (0.21-0.71)	0.42 (0.18-0.7)	-1.68 (-1.92--1.44)
Djibouti	0.74 (0.31-1.22)	1.52 (0.63-2.75)	1.05	0.18 (0.08-0.3)	0.12 (0.05-0.22)	-3.05 (-3.34--2.76)
Dominica	0.03 (0.01-0.05)	0.03 (0.01-0.05)	0	0.04 (0.02-0.07)	0.04 (0.02-0.07)	-1.37 (-1.81--0.93)
Dominican Republic	6.57 (2.92-10.51)	13.38 (5.89-21.23)	1.04	0.09 (0.04-0.15)	0.12 (0.05-0.19)	-1.49 (-1.85--1.12)
Ecuador	9.77 (4.28-15.15)	55.48 (23.17-88.74)	4.68	0.1 (0.04-0.15)	0.31 (0.13-0.49)	2.28 (1.7-2.87)
Egypt	2.48 (1.04-4.29)	3.96 (1.43-7.59)	0.6	0 (0-0.01)	0 (0-0.01)	-0.26 (-0.81-0.29)
El Salvador	0.5 (0.22-0.82)	1.46 (0.57-2.61)	1.92	0.01 (0-0.02)	0.02 (0.01-0.04)	1.31 (1.14-1.48)
Equatorial Guinea	0.21 (0.08-0.37)	0.1 (0.03-0.19)	-0.52	0.05 (0.02-0.09)	0.01 (0-0.01)	-7.06 (-8.17--5.94)

Eritrea	0.62 (0.24-1.13)	1.17 (0.47-2.17)	0.89	0.02 (0.01-0.03)	0.02 (0.01-0.03)	-0.44 (-0.65--0.22)
Estonia	2.36 (1.06-3.72)	2.27 (0.99-4.01)	-0.04	0.15 (0.07-0.24)	0.17 (0.08-0.31)	-2.43 (-2.76--2.09)
Ethiopia	17.33 (7.22-28.79)	11.37 (4.67-20.08)	-0.34	0.03 (0.01-0.06)	0.01 (0-0.02)	-4.93 (-5.26--4.61)
Fiji	0.23 (0.1-0.38)	0.19 (0.07-0.34)	-0.17	0.03 (0.01-0.05)	0.02 (0.01-0.04)	-2.4 (-3.06--1.73)
Finland	17.58 (8.03-27.9)	18.93 (7.75-32.15)	0.08	0.35 (0.16-0.56)	0.34 (0.14-0.58)	-2.57 (-2.86--2.27)
France	351.42 (163.24-552.45)	421.79 (183.14-690.92)	0.2	0.61 (0.28-0.96)	0.64 (0.28-1.04)	-1.27 (-1.35--1.19)
Gabon	0.4 (0.15-0.73)	0.53 (0.22-0.9)	0.32	0.04 (0.02-0.07)	0.03 (0.01-0.05)	-0.96 (-1.06--0.86)
Georgia	3.38 (1.51-5.5)	6.24 (2.7-10.31)	0.85	0.06 (0.03-0.1)	0.17 (0.07-0.29)	2.38 (1.87-2.89)
Germany	385.21 (181.47-623.26)	355.98 (150.62-584.05)	-0.08	0.48 (0.23-0.78)	0.42 (0.18-0.68)	-2.5 (-2.75--2.25)
Ghana	0.42 (0.16-0.73)	0.04 (0.01-0.12)	-0.9	0 (0-0)	0 (0-0)	-10.48 (-11.66--9.29)
Greece	10.47 (4.55-17.32)	29.19 (12.6-49.42)	1.79	0.1 (0.04-0.17)	0.29 (0.12-0.49)	1.03 (0.76-1.3)
Greenland	0.16 (0.07-0.27)	0.11 (0.04-0.19)	-0.31	0.29 (0.12-0.48)	0.2 (0.08-0.34)	-3.83 (-3.99--3.67)
Grenada	0.14 (0.06-0.22)	0.18 (0.08-0.3)	0.29	0.16 (0.07-0.26)	0.18 (0.08-0.29)	0.23 (-0.02-0.48)
Guam	0 (0-0)	0 (0-0)	NA	0 (0-0)	0 (0-0)	-3.45 (-3.83--3.08)
Guatemala	0.32 (0.13-0.55)	2.1 (0.86-3.6)	5.56	0 (0-0.01)	0.01 (0.01-0.02)	0.99 (0.36-1.

						63)
Guinea	0.76 (0.33-1.24)	1.3 (0.54-2.3)	0.71	0.01 (0.01-0.02)	0.01 (0-0.02)	0.16 (-0.06-0.38)
Guinea-Bissau	1.26 (0.52-1.99)	0.64 (0.25-1.1)	-0.49	0.13 (0.05-0.2)	0.03 (0.01-0.05)	-4.2 (-4.62--3.79)
Guyana	1.42 (0.63-2.2)	1.07 (0.44-1.74)	-0.25	0.18 (0.08-0.28)	0.14 (0.06-0.23)	-1.91 (-2.32--1.49)
Haiti	3.2 (1.31-5.44)	10.61 (4.63-18.74)	2.32	0.05 (0.02-0.09)	0.08 (0.04-0.15)	0.43 (0.04-0.82)
Honduras	0.32 (0.13-0.54)	1.73 (0.7-3)	4.41	0.01 (0-0.01)	0.02 (0.01-0.03)	1.86 (1.47-2.25)
Hungary	47.23 (20.19-74)	71.64 (31.38-113.78)	0.52	0.45 (0.19-0.71)	0.75 (0.33-1.19)	-0.27 (-0.76-0.23)
Iceland	1.45 (0.64-2.26)	1.84 (0.8-3)	0.27	0.57 (0.25-0.89)	0.52 (0.23-0.86)	-1.69 (-1.93--1.44)
India	367.67 (169.32-578.97)	605.35 (264.07-924.55)	0.65	0.04 (0.02-0.07)	0.04 (0.02-0.07)	-1.67 (-1.93--1.42)
Indonesia	387.41 (169.36-612.05)	712.48 (310.45-1166.17)	0.84	0.21 (0.09-0.33)	0.26 (0.11-0.42)	-0.71 (-0.86--0.56)
Iran	11.92 (5.59-19.33)	20.58 (8.65-33.42)	0.73	0.02 (0.01-0.03)	0.02 (0.01-0.04)	-1.49 (-1.92--1.05)
Iraq	1.45 (0.62-2.43)	12.56 (5.38-21.13)	7.66	0.01 (0-0.01)	0.03 (0.01-0.05)	3.09 (2.33-3.85)
Ireland	7.37 (3.35-11.8)	8.29 (3.42-14.27)	0.12	0.2 (0.09-0.33)	0.17 (0.07-0.29)	-1.34 (-1.6--1.07)
Israel	3.47 (1.46-5.97)	5.82 (2.47-10.42)	0.68	0.07 (0.03-0.12)	0.06 (0.03-0.11)	-2.08 (-2.25--1.92)
Italy	179.43 (79.61-278.6)	271.25 (116.23-418.44)	0.51	0.32 (0.14-0.49)	0.45 (0.19-0.7)	-0.47 (-0.74--0.21)
Jamaica	1.26	3.09	1.45	0.05	0.11 (0.04-0.2)	1.57

	(0.57-2.12)	(1.15-5.49)		(0.02-0.09)		(1.3-1.85)
Japan	290.69 (122.24-452.91)	824.51 (359.64-1336.77)	1.84	0.23 (0.1-0.36)	0.65 (0.28-1.05)	0.41 (0.26-0.56)
Jordan	1.81 (0.8-2.85)	5.72 (2.39-9.5)	2.16	0.05 (0.02-0.08)	0.05 (0.02-0.08)	-2.3 (-2.5--2.1)
Kazakhstan	23.28 (9.48-36.11)	14.29 (6.67-23.29)	-0.39	0.14 (0.06-0.22)	0.08 (0.04-0.12)	-3.34 (-4.13--2.53)
Kenya	1.72 (0.73-2.83)	4.36 (1.99-7.27)	1.53	0.01 (0-0.01)	0.01 (0-0.01)	0.22 (0.05-0.39)
Kiribati	0.01 (0.01-0.02)	0.02 (0.01-0.04)	1	0.02 (0.01-0.03)	0.02 (0.01-0.03)	-1.11 (-1.25--0.97)
Kuwait	0.35 (0.16-0.56)	1.57 (0.65-2.56)	3.49	0.02 (0.01-0.03)	0.03 (0.01-0.06)	0.05 (-0.39-0.49)
Kyrgyzstan	3.72 (1.64-5.88)	2.19 (0.98-3.49)	-0.41	0.08 (0.04-0.13)	0.03 (0.01-0.05)	-3.89 (-4.34--3.45)
Lao People's Democratic Republic	23.28 (8.7-38)	29.52 (11.87-49.97)	0.27	0.56 (0.21-0.91)	0.4 (0.16-0.68)	-2.06 (-2.18--1.94)
Latvia	6.04 (2.64-9.58)	7.1 (2.93-11.4)	0.18	0.23 (0.1-0.36)	0.38 (0.16-0.61)	-0.74 (-1.29--0.19)
Lebanon	0.46 (0.18-0.83)	2 (0.79-3.55)	3.35	0.02 (0.01-0.03)	0.04 (0.01-0.06)	2.05 (1.47-2.62)
Lesotho	0.05 (0.02-0.09)	0.06 (0.02-0.12)	0.2	0 (0-0.01)	0 (0-0.01)	0.28 (0.12-0.44)
Liberia	0.88 (0.41-1.42)	2.93 (1.26-5.52)	2.33	0.04 (0.02-0.06)	0.05 (0.02-0.1)	1.64 (1.3-1.98)
Libya	1 (0.45-1.67)	4.81 (2.03-8.03)	3.81	0.02 (0.01-0.04)	0.07 (0.03-0.12)	1.87 (1.55-2.19)
Lithuania	1.88 (0.81-3.25)	4.06 (1.73-6.99)	1.16	0.05 (0.02-0.09)	0.15 (0.06-0.26)	1.48 (0.95-2.02)

Luxembourg	2.21 (1.01-3.4)	2.17 (0.87-3.71)	-0.02	0.58 (0.27-0.89)	0.34 (0.13-0.58)	-2.54 (-2.79--2.28)
Macedonia	1.89 (0.85-2.96)	3.33 (1.38-5.54)	0.76	0.09 (0.04-0.15)	0.15 (0.06-0.25)	-0.37 (-0.9-0.16)
Madagascar	4.59 (2.01-7.45)	10.17 (4.19-16.82)	1.22	0.04 (0.02-0.06)	0.04 (0.01-0.06)	0.01 (-0.13-0.15)
Malawi	0.13 (0.05-0.24)	0.17 (0.06-0.36)	0.31	0 (0-0)	0 (0-0)	-2.68 (-3.22--2.13)
Malaysia	72.03 (33.39-113.39)	129.04 (57.78-202.46)	0.79	0.41 (0.19-0.64)	0.41 (0.18-0.64)	-1.61 (-1.82--1.41)
Maldives	0.15 (0.06-0.24)	0.38 (0.16-0.61)	1.53	0.07 (0.03-0.11)	0.07 (0.03-0.12)	-2.2 (-2.58--1.82)
Mali	1.31 (0.6-2.07)	0.94 (0.34-1.69)	-0.28	0.02 (0.01-0.02)	0 (0-0.01)	-3.71 (-4.26--3.16)
Malta	0.9 (0.4-1.43)	1.06 (0.45-1.78)	0.18	0.24 (0.11-0.39)	0.24 (0.1-0.4)	-1.84 (-2.39--1.3)
Marshall Islands	0 (0-0.01)	0.01 (0-0.01)	Inf	0.01 (0-0.02)	0.01 (0-0.03)	-0.91 (-0.99--0.83)
Mauritania	0.6 (0.25-0.93)	0.57 (0.24-1.02)	-0.05	0.03 (0.01-0.05)	0.01 (0.01-0.02)	-2.38 (-2.8--1.95)
Mauritius	2.75 (1.24-4.08)	3.93 (1.7-6.18)	0.43	0.25 (0.11-0.37)	0.31 (0.13-0.49)	-2.62 (-3.14--2.09)
Mexico	4.15 (1.73-7.28)	22.58 (9.73-39.44)	4.44	0 (0-0.01)	0.02 (0.01-0.03)	2.06 (1.66-2.47)
Micronesia (Federated States of)	0.02 (0.01-0.03)	0.02 (0.01-0.03)	0	0.02 (0.01-0.03)	0.02 (0.01-0.03)	-0.97 (-1.06--0.88)
Moldova	2.5 (1.04-4.2)	5.59 (2.35-9.78)	1.24	0.06 (0.02-0.09)	0.16 (0.07-0.27)	1.47 (1.08-1.86)
Monaco	0.06 (0.02-0.1)	0.06 (0.02-0.11)	0	0.18 (0.07-0.34)	0.16 (0.06-0.28)	-0.61 (-0.85--

						0.38)
Mongolia	5.8 (2.38-9.2)	7.66 (3.28-12.1)	0.32	0.27 (0.11-0.43)	0.23 (0.1-0.36)	-2.07 (-2.28--1.86)
Montenegro	0.4 (0.19-0.65)	0.75 (0.28-1.26)	0.87	0.06 (0.03-0.1)	0.12 (0.05-0.2)	-0.33 (-0.86-0.21)
Morocco	0.53 (0.2-1.03)	1.27 (0.39-2.57)	1.4	0 (0-0)	0 (0-0.01)	-0.16 (-0.28--0.04)
Mozambique	1.9 (0.82-3.04)	1.46 (0.6-2.48)	-0.23	0.01 (0.01-0.02)	0 (0-0.01)	-2.74 (-3.17--2.31)
Myanmar	227.87 (93.8-384.89)	229.09 (101.52-367.94)	0.01	0.56 (0.23-0.95)	0.41 (0.18-0.65)	-2.95 (-3.21--2.68)
Namibia	0.56 (0.25-0.9)	1.17 (0.52-1.95)	1.09	0.04 (0.02-0.06)	0.05 (0.02-0.08)	-0.13 (-0.25--0.02)
Nauru	0 (0-0)	0 (0-0)	NA	0 (0-0.01)	0.01 (0-0.01)	0.6 (-0.7-1.91)
Nepal	9.07 (3.83-15.46)	6.86 (2.87-11.83)	-0.24	0.05 (0.02-0.08)	0.02 (0.01-0.04)	-3.89 (-4.51--3.25)
Netherlands	103.6 (45.61-158.81)	98.51 (42.97-163.88)	-0.05	0.69 (0.31-1.06)	0.57 (0.25-0.95)	-2.42 (-2.69--2.14)
New Zealand	13.77 (6.02-22.44)	20.63 (8.52-34.45)	0.5	0.4 (0.18-0.66)	0.4 (0.16-0.67)	-0.66 (-0.94--0.38)
Nicaragua	0.89 (0.38-1.37)	2.04 (0.85-3.34)	1.29	0.02 (0.01-0.04)	0.03 (0.01-0.05)	-1.79 (-2.08--1.49)
Niger	0.74 (0.33-1.21)	0.92 (0.37-1.72)	0.24	0.01 (0-0.02)	0 (0-0.01)	-3.2 (-3.63--2.76)
Nigeria	7.72 (3.33-12.91)	5.23 (2.01-9.07)	-0.32	0.01 (0-0.01)	0 (0-0)	-3.57 (-3.97--3.16)
Niue	0 (0-0)	0 (0-0)	NA	0.01 (0.01-0.03)	0.01 (0-0.02)	-2.14 (-2.31--1.97)
Northern	0 (0-0)	0 (0-0)	NA	0 (0-0)	0 (0-0.01)	1.75

Mariana Islands						(1.21-2.29)
Norway	35.16 (16.42-54.15)	29.45 (12.79-47.98)	-0.16	0.83 (0.39-1.28)	0.54 (0.24-0.89)	-2.48 (-2.65--2.31)
Oman	0.25 (0.11-0.41)	0.23 (0.09-0.38)	-0.08	0.01 (0.01-0.02)	0 (0-0.01)	-3.02 (-3.66--2.38)
Pakistan	82.31 (37.85-130.26)	181.7 (81.24-293.02)	1.21	0.07 (0.03-0.12)	0.08 (0.03-0.12)	0.09 (-0.17-0.35)
Palau	0 (0-0)	0 (0-0)	NA	0.01 (0-0.02)	0.01 (0-0.02)	-1.36 (-1.54--1.17)
Palestine	3.71 (1.71-6.47)	8.87 (3.91-14.79)	1.39	0.18 (0.08-0.32)	0.17 (0.08-0.29)	-0.13 (-0.46-0.2)
Panama	5.38 (2.36-8.14)	16.81 (7.04-26.5)	2.12	0.23 (0.1-0.34)	0.39 (0.16-0.62)	0.09 (-0.19-0.36)
Papua New Guinea	0.08 (0.03-0.15)	0.17 (0.07-0.33)	1.12	0 (0-0)	0 (0-0)	-1.16 (-1.34--0.98)
Paraguay	0.48 (0.2-0.8)	1.8 (0.71-3.12)	2.75	0.01 (0-0.02)	0.03 (0.01-0.04)	1.36 (1.14-1.58)
Peru	20.91 (9.1-33.21)	17.14 (7.37-30)	-0.18	0.1 (0.04-0.15)	0.05 (0.02-0.08)	-4.87 (-5.44--4.31)
Philippines	157.03 (70.76-240.97)	518.43 (239.16-809.88)	2.3	0.25 (0.11-0.38)	0.46 (0.21-0.72)	0.65 (0.47-0.82)
Poland	41.6 (18.3-66.45)	82.05 (31.68-138.85)	0.97	0.11 (0.05-0.17)	0.21 (0.08-0.36)	0.17 (-0.23-0.56)
Portugal	21.58 (9.76-34.33)	41.54 (18.35-71.06)	0.92	0.21 (0.1-0.34)	0.39 (0.17-0.67)	0.11 (-0.17-0.4)
Puerto Rico	7.17 (3.05-11.78)	10.1 (4.19-17.46)	0.41	0.2 (0.08-0.33)	0.31 (0.13-0.53)	-1.52 (-1.62--1.41)
Qatar	0.01 (0.01-0.03)	0.05 (0.02-0.09)	4	0 (0-0.01)	0 (0-0)	-3.75 (-4.26--3.24)

Romania	22.64 (9.72-36.63)	28 (11.02-48.4)	0.24	0.1 (0.04-0.16)	0.15 (0.06-0.26)	-1.39 (-1.73--1.05)
Russian Federation	287.28 (132.24-440.97)	459.3 (214.6-720.88)	0.6	0.19 (0.09-0.29)	0.32 (0.15-0.5)	-0.74 (-1.36--0.11)
Rwanda	0.03 (0.01-0.07)	0.07 (0.02-0.18)	1.33	0 (0-0)	0 (0-0)	-2.99 (-4.04--1.93)
Saint Kitts and Nevis	0.39 (0.17-0.6)	0.6 (0.26-0.96)	0.54	0.94 (0.42-1.44)	1.02 (0.44-1.63)	0.52 (0.33-0.71)
Saint Lucia	0.14 (0.06-0.23)	0.61 (0.26-1.01)	3.36	0.11 (0.05-0.17)	0.34 (0.14-0.57)	1.15 (0.9-1.4)
Saint Vincent and the Grenadines	0.14 (0.06-0.22)	0.23 (0.1-0.37)	0.64	0.13 (0.06-0.2)	0.2 (0.09-0.32)	-1.39 (-1.67--1.11)
Samoa	0 (0-0.01)	0 (0-0.01)	NA	0 (0-0)	0 (0-0)	-2.73 (-3.42--2.05)
San Marino	0.08 (0.03-0.14)	0.09 (0.04-0.16)	0.12	0.35 (0.15-0.61)	0.28 (0.12-0.5)	-1.65 (-2.16--1.14)
Sao Tome and Principe	0.01 (0-0.01)	0.01 (0-0.01)	0	0 (0-0.01)	0 (0-0)	-0.55 (-1.22-0.13)
Saudi Arabia	1.35 (0.6-2.29)	4.69 (1.83-7.99)	2.47	0.01 (0-0.01)	0.01 (0-0.02)	0.52 (0.17-0.86)
Senegal	2.41 (1.06-3.78)	0.87 (0.34-1.61)	-0.64	0.03 (0.01-0.05)	0.01 (0-0.01)	-6.4 (-7.3--5.5)
Serbia	22.99 (10.16-35.98)	32.91 (12.7-55.86)	0.43	0.24 (0.11-0.37)	0.37 (0.14-0.63)	-1.67 (-2.06--1.28)
Seychelles	0.24 (0.11-0.38)	0.25 (0.12-0.42)	0.04	0.33 (0.15-0.53)	0.24 (0.12-0.4)	-1.52 (-1.66--1.39)
Sierra Leone	6.01 (2.77-9.34)	6.44 (2.66-10.62)	0.07	0.14 (0.07-0.22)	0.07 (0.03-0.12)	-1.59 (-1.69--1.48)
Singapore	10.42 (4.72-16.83)	17.23 (7.22-28.19)	0.65	0.34 (0.15-0.55)	0.3 (0.13-0.49)	-3.01 (-3.18--2.83)

Slovakia	24.35 (11.1-37.83)	50.99 (20.66-82.42)	1.09	0.46 (0.21-0.72)	0.94 (0.38-1.52)	0.91 (0.33-1.51)
Slovenia	5.52 (2.38-8.79)	6.77 (2.87-11.37)	0.23	0.28 (0.12-0.45)	0.33 (0.14-0.55)	-1.91 (-2.09--1.73)
Solomon Islands	0.01 (0-0.02)	0.02 (0.01-0.05)	1	0 (0-0.01)	0 (0-0.01)	-0.29 (-0.47--0.1)
Somalia	7.37 (3.11-13.29)	24.78 (10.91-44.04)	2.36	0.09 (0.04-0.17)	0.11 (0.05-0.2)	0.69 (0.31-1.07)
South Africa	6.98 (3.1-11.14)	23.87 (10.55-37.61)	2.42	0.02 (0.01-0.03)	0.04 (0.02-0.07)	1.55 (1.29-1.81)
South Korea	81.7 (34.73-128.46)	480.54 (218.04-806.63)	4.88	0.18 (0.08-0.29)	0.93 (0.42-1.56)	1.19 (0.82-1.56)
South Sudan	0.32 (0.11-0.68)	0.5 (0.18-0.96)	0.56	0.01 (0-0.01)	0.01 (0-0.01)	-0.04 (-0.16-0.09)
Spain	97.79 (43.97-154.07)	240.86 (102.73-399.9)	1.46	0.25 (0.11-0.4)	0.53 (0.23-0.88)	1.06 (0.78-1.35)
Sri Lanka	5.51 (2.56-8.39)	7.53 (2.85-12.8)	0.37	0.03 (0.01-0.05)	0.03 (0.01-0.06)	-2.13 (-2.44--1.83)
Sudan	10.77 (4.24-19.76)	17.2 (6.67-31)	0.6	0.05 (0.02-0.1)	0.04 (0.02-0.07)	-1.1 (-1.16--1.04)
Suriname	1.63 (0.72-2.47)	4.09 (1.78-6.66)	1.51	0.42 (0.19-0.64)	0.71 (0.31-1.15)	0.15 (-0.14-0.43)
Swaziland	0.16 (0.06-0.27)	0.54 (0.21-0.95)	2.38	0.02 (0.01-0.03)	0.05 (0.02-0.08)	2.23 (1.64-2.83)
Sweden	62.37 (28.75-97.01)	46.15 (18.59-77.07)	-0.26	0.73 (0.33-1.13)	0.44 (0.18-0.74)	-2.76 (-2.95--2.57)
Switzerland	36.44 (15.97-56.61)	50.9 (22.41-83.97)	0.4	0.53 (0.23-0.82)	0.57 (0.25-0.94)	-0.96 (-1.29--0.62)
Syria	2.52 (1.04-4.02)	6.28 (2.58-10.72	1.49	0.02 (0.01-0.03)	0.04 (0.02-0.08)	-0.43 (-0.82--

		)				0.04)
Taiwan (Province of China)	63.67 (28.85-97.69)	151.83 (65.3-239.05)	1.38	0.31 (0.14-0.48)	0.64 (0.28-1.01)	-0.23 (-0.44--0.02)
Tajikistan	5.93 (2.64-9.1)	6.14 (2.6-10.07)	0.04	0.11 (0.05-0.17)	0.06 (0.03-0.1)	-3.08 (-3.49--2.66)
Tanzania	2.17 (0.9-3.75)	4.4 (1.81-7.8)	1.03	0.01 (0-0.01)	0.01 (0-0.01)	-0.87 (-1.1--0.63)
Thailand	306.29 (138.57-481.1)	703.95 (296.11-1186.27)	1.3	0.54 (0.24-0.85)	1.06 (0.44-1.78)	-1.36 (-1.56--1.17)
The Bahamas	0.61 (0.26-0.91)	2 (0.93-3.21)	2.28	0.24 (0.1-0.36)	0.52 (0.24-0.83)	0.83 (0.58-1.09)
The Gambia	0.28 (0.12-0.43)	0.18 (0.07-0.29)	-0.36	0.03 (0.01-0.04)	0.01 (0-0.01)	-5.47 (-6.25--4.68)
Timor-Leste	0.34 (0.14-0.55)	1.02 (0.43-1.65)	2	0.04 (0.02-0.07)	0.07 (0.03-0.12)	-0.32 (-0.63--0.02)
Togo	0.08 (0.03-0.15)	0.17 (0.06-0.34)	1.12	0 (0-0)	0 (0-0)	-1.15 (-1.61--0.69)
Tokelau	0 (0-0)	0 (0-0)	NA	0.02 (0.01-0.04)	0.01 (0-0.02)	-3.06 (-3.17--2.95)
Tonga	0.01 (0-0.01)	0.01 (0-0.02)	0	0.01 (0-0.01)	0.01 (0-0.02)	-1.18 (-1.27--1.09)
Trinidad and Tobago	2.48 (1.06-3.83)	3.24 (1.4-5.33)	0.31	0.21 (0.09-0.32)	0.23 (0.1-0.38)	-3.03 (-3.39--2.68)
Tunisia	1.08 (0.44-1.81)	1.98 (0.74-3.66)	0.83	0.01 (0.01-0.02)	0.02 (0.01-0.03)	-2.11 (-2.35--1.88)
Turkey	6.61 (2.57-12.12)	14.32 (5.29-27.46)	1.17	0.01 (0-0.02)	0.02 (0.01-0.03)	-0.25 (-0.44--0.06)
Turkmenistan	3.21 (1.43-4.92)	2.46 (1.1-4.21)	-0.23	0.09 (0.04-0.13)	0.05 (0.02-0.08)	-4.05 (-4.49--3.6)
Tuvalu	0 (0-0)	0 (0-0)	NA	0.02	0.02	-1.66

				(0.01-0.04)	(0.01-0.03)	(-1.84--1.47)
Uganda	0.78 (0.29-1.4)	2.11 (0.73-3.77)	1.71	0 (0-0.01)	0 (0-0.01)	0.06 (-0.12-0.24)
Ukraine	72.2 (31.55-114.02)	82.34 (34.85-142.84)	0.14	0.14 (0.06-0.22)	0.19 (0.08-0.33)	-1.05 (-1.64--0.45)
United Arab Emirates	0.25 (0.09-0.44)	1.24 (0.47-2.2)	3.96	0.01 (0-0.02)	0.01 (0-0.02)	2.31 (1.58-3.05)
United Kingdom	462.76 (203.96-750.44)	359.05 (164.03-577.47)	-0.22	0.81 (0.36-1.31)	0.53 (0.24-0.85)	-2.07 (-2.4--1.75)
United States	1440.35 (662.8-2215.74)	913.9 (406.96-1442.85)	-0.37	0.57 (0.26-0.87)	0.27 (0.12-0.43)	-3.46 (-3.65--3.27)
Uruguay	29.07 (13.18-44)	19.63 (8.45-31.98)	-0.32	0.93 (0.42-1.4)	0.58 (0.25-0.94)	-3.09 (-3.29--2.9)
Uzbekistan	14.18 (6.14-21.87)	9.54 (4.01-15.54)	-0.33	0.07 (0.03-0.1)	0.03 (0.01-0.05)	-4.99 (-5.57--4.41)
Vanuatu	0.01 (0-0.02)	0.02 (0.01-0.03)	1	0.01 (0-0.01)	0.01 (0-0.01)	-2.39 (-2.64--2.14)
Venezuela	13.66 (5.94-20.31)	56.16 (24.53-94.36)	3.11	0.07 (0.03-0.11)	0.21 (0.09-0.35)	0.49 (0.19-0.79)
Viet Nam	284.25 (124.76-438.98)	682.91 (306.36-1113.48)	1.4	0.42 (0.18-0.64)	0.68 (0.31-1.11)	-0.14 (-0.31-0.04)
Virgin Islands, U.S.	0.23 (0.09-0.38)	0.2 (0.08-0.35)	-0.13	0.22 (0.08-0.36)	0.23 (0.09-0.4)	-3.03 (-3.41--2.66)
Yemen	4.66 (2.03-7.9)	18.29 (7.86-34.05)	2.92	0.03 (0.01-0.06)	0.05 (0.02-0.1)	0.13 (-0.18-0.45)
Zambia	0.55 (0.22-0.99)	2.18 (0.75-4.79)	2.96	0.01 (0-0.01)	0.01 (0-0.02)	1.24 (0.73-1.76)
Zimbabwe	1.59 (0.7-2.62)	3.01 (1.19-5.15)	0.89	0.02 (0.01-0.03)	0.02 (0.01-0.03)	0.01 (-0.41-0.43)

Supplementary table 2. The deaths of CRC-LFD cases and rates in 1990 and 2021 across 204 countries, and the trends from 1990 to 2021.
